# Performance optimization and machine learning-guided parameter sensitivity analysis of lead-free KGeCl_3_ perovskite solar cells

**DOI:** 10.1039/d6ra00262e

**Published:** 2026-02-13

**Authors:** Tanzir Ahamed, Md. Mehedi Hasan Bappy, Mohammad Rahimul Islam, Md. Shihab Uddin, Md. Arafat Hossain, Tanvir Ahammed

**Affiliations:** a Department of Electrical and Electronic Engineering, CCN University of Science and Technology Cumilla-3503 Bangladesh tanzir.eee2k15@gmail.com; b Department of Electrical and Electronic Engineering, Chittagong University of Engineering and Technology Chattogram-4349 Bangladesh; c Department of Computer Science and Engineering, Daffodil International University Dhaka-1216 Bangladesh su130776@gmail.com; d NanoBio Technology Center, Daffodil International University Dhaka-1216 Bangladesh; e Department of Information and Communication Engineering, Bangladesh Army University of Engineering & Technology Natore-6431 Bangladesh; f Department of Materials Science and Engineering, University of Rajshahi Rajshahi-6205 Bangladesh

## Abstract

This study gives a realistic insight into the effectiveness of lead-free Ge-based perovskite solar cells (PSCs) using KGeCl_3_ as the absorber layer in combination with four different electron transport layers (ETLs), including WS_2_, ZnSe, PC_60_BM, and SnS_2_, with copper iron tin sulfide (CFTS) serving as the hole transport layer (HTL). Initially, key material parameters such as layer thickness, donor density (*N*_D_), acceptor density (*N*_A_), defect density (*N*_t_), interface defect densities (IL1 & IL2), series resistance (*R*_s_), shunt resistance (*R*_sh_), operating temperature (K), and back contact work function (eV) are varied using a SCAPS-1D simulator to optimize device performance. Between the four cell configurations, the FTO/CFTS/KGeCl_3_/WS_2_/Au structure has achieved the highest performance with a power conversion efficiency (PCE) of 21.39%, short-circuit current density (*J*_SC_) of 39.526 mA cm^−2^, fill factor (FF) of 76.56%, and open-circuit voltage (*V*_OC_) of 0.706 V at a simulated temperature of 300 K. Other configurations using ZnSe, PC_60_BM, and SnS_2_ as ETLs showed PCE values of 21.38%, 21.05%, and 20.43%, respectively. Furthermore, an integrated machine learning framework with four supervised learning methods, *i.e.*, Random Forest, XGBoost, CatBoost, and Decision Tree, has been utilized to effectively evaluate the importance of material features. Out of the algorithms, CatBoost has the highest performance with *R*^2^ and accuracy values of 0.984 and 99.344%, respectively.

## Abstract 2

Lead-free KGeCl_3_ perovskite solar cells are optimized using SCAPS-1D and machine learning with WS_2_, ZnSe, PC_60_BM, and SnS_2_ ETLs. The ETL WS_2_ provides the best band alignment, lowest recombination, and highest simulated efficiency.

## Graphical abstract

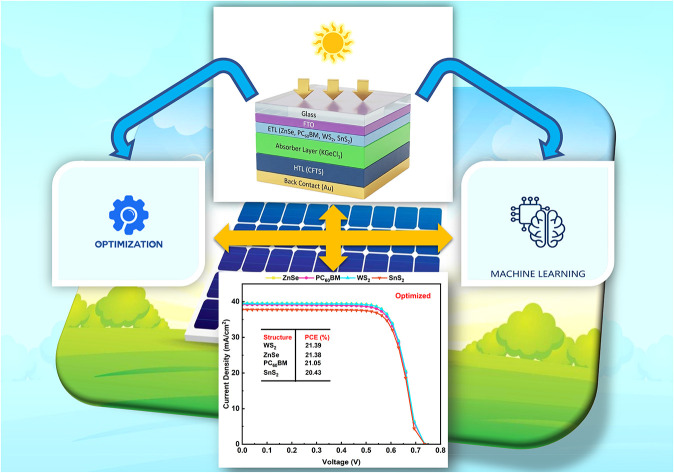

## 1. Introduction

With the global energy requirement and the depletion of fossil fuel reserves, as well as the deep impact of the carbon-based energy environment, research into sustainable, efficient photovoltaic (PV) technologies has become very popular.^1^ In the present context, perovskite solar cells (PSCs) could be considered as being among the very promising next-generation photovoltaic structures.^2,3^ Since their first demonstration in 2009, the power conversion efficiency (PCE) of PSCs has increased at an unprecedented rate, rising from 3.8% to over 26.1% within less than ten years, and with reports of even higher performance under optimized laboratory conditions.^4^ The sides of perovskite cells are evolving quickly; as a result, considerable research has been done on morphology control.^5^ Such rapid advances are due mostly to the exceptional optoelectronic features of perovskite materials; they can be fine-tuned, have strong absorption coefficients, long charge-carrier diffusion paths, and high electron–hole mobilities.^6–9^ In addition, PSCs benefit from facile, low-cost manufacture as well as compatibility with flexible substrates, all of which make them a product attractive for industrial production and scale-up.^8,10^

Perovskite materials have set a landmark to make a gradual improvement in advancing the PCE of PSCs.^11^ A diverse amount of perovskite has been developed through bandgap engineering over the last few decades.^12^ ABX_3_ contains the basic structure of perovskite that includes A, B, and X for organic inorganic cations, metal cation, and halogen ion, respectively.^13^ Recently, various kinds of these structures with different compositions have been promising for use as a solar absorber. It has been found that through the addition of small cations such as cesium (Cs^+^), methylammonium (MA^+^), or formamide (FA^+^), with halide ions, perovskite structures can be modified and have their bandgaps adjusted towards different light spectra.^14–16^ At present, however, the industry is faced with a shortage of raw materials and increasing demand for end-use products after so many years of pure research.^10^ Consequently, there is a growing need that lead-halide perovskite sensitized cells will soon appear on the market.^17^ But, lead-based perovskite has a major drawback because of its toxic nature for the environment.^18^ One of the leading candidates for replacement of both lead-free necessity and environmental concerns is germanium (Ge^2+^).^19^ Ge^2+^ has properties that make it much more resistant than tin (Sn^2+^) to oxidize and also happens to be able to exhibit suitable direct bandgaps with high absorption coefficients (∼10^5^ cm^−1^) and an elevated static refractive index.^20,21^ Most importantly, germanium-based compounds are more friendly to the environment than their heavy metal counterparts like lead, and the element has relatively abundant natural reserves.^22^ Its usage happens frequently in sustainable energy applications; however, it has had a history of low performance because of the compound's migration up into Ge^4+^, lower defect tolerance, and sensitivity to moisture.^23^ On top of that, Shockley–Read–Hall (SRH) recombination is sped up and device lifetimes are shortened as a consequence.^24^ At the moment, potassium germanium chloride (KGeCl_3_) is making a name for itself as a next-generation lead-free perovskite material because of its superb optoelectronic characteristics with good stability, both thermal and mechanical.^25,26^ After crystallizing with K^+^ as the A-site, Ge^2+^ as the B-site, and Cl^−^ on the third X-site into perovskite-structured ABX_3_ compounds, KGeCl_3_ has been found to have excellent thermal resistance, remaining stable and showing no decomposition at over 100 °C.^27^ Meanwhile, it offers greater tear strength in humid environments than organic cation-based perovskites and shows much less deterioration from UV rays.^23^ A past report by Roknuzzaman *et al.* in ref. 28 revealed that CsBX_3_, where B stands for Sn, Ge, and X for Cl, Br, and I-based perovskite, has more magnificent optoelectronic properties than the Pb-based perovskite for solar cell applications. Its single-junction quasi-bandgap energy is about 1.1 eV, so it lies in the optimal range for solar cells.^29^ It also has a strong absorption coefficient and will harvest photons efficiently within the visible spectrum. Furthermore, KGeCl_3_ demonstrates low rates for Auger recombination (<10^−8^ cm^3^ s^−1^) and high charge carrier mobilities of 60–100 cm^2^ V^−1^ s^−1^.^30^ In other words, quick charge transport with a minimum loss in energy is assured. Together with favorable band alignments with a vast variety of electron and hole transport layers, these intrinsic material properties have already led to simulated device conversion efficiencies (PCEs) of higher than 25% when the assembly of layers is optimized. A recent study by Ur Rehman *et al.* showed a massive improvement *via* numerical simulation by FTO/MoO_3_/KGeCl_3_/WS_2_/Au structure with PCE of 29.83% with continuous optimization of every layer.^31^ This advancement is possible due to selecting the efficient carrier transport layers (CTLs), including the ETL and HTL. Taken together, these attributes make KGeCl_3_ an environmentally friendly, structurally stable, and high-performance absorber material—a green candidate for next-generation PSCs and sustainable PV technologies.

Due to their unique optoelectronic properties and excellent energy band alignment, inorganic and organic semiconductors have been intended for use in electron transport layers (ETLs) in perovskite solar cells.^32^ A careful selection of the CTLs can enhance both stability and performance of solar cells. Transition metal dichalcogenides like WS_2_ offer high electron mobility and strong environmental stability.^33^ Proper band alignment between the absorber layer is one of the main motives for choosing the CTLs.^34^ This also ensures minimum parasitic resistance and lower recombination loss.^35^ With atomically thin layered structures, they provide efficient charge extraction while retaining transparency to sunlight enough for an absorbing layer made up of the perovskite crystal.^36^ ZnSe is a wide bandgap (≈2.7 eV) semiconductor with high transparency and chemical stability.^37^ The conduction band alignment with perovskites makes it an environmentally friendly, low-cost, and non-toxic ETL alternative to traditional materials such as TiO_2_. For this similar reason, fullerene derivatives like PC_60_BM can be found in many perovskite structures. They are easily processed at low temperatures, passivate the interface traps, and have a strong attraction for electrons. Those phenomena greatly reduce hysteresis and greatly improve device reproducibility.^38,39^ In addition, metal chalcogenides with layer structures, such as SnS_2_, share a high electron mobility with a wide bandgap (≈2.2 eV).^40^ They are nontoxic, environmentally friendly, and durable. As a result, they help ensure long-term device stability through efficient charge transport.^34^ Compared with conventional TiO_2_ and SnO_2_, the ETL materials WS_2_, ZnSe, SnS_2_, and PC_60_BM offer more favorable band alignment with KGeCl_3_, reduced interfacial recombination, and enhanced electron extraction.^41,42^ Those phenomena are maintaining high optical transparency, environmental compatibility, and efficient charge transport.^43^ Approaches with KGeCl_3_ with distinct carrier transport layers (CTLs) have made PCEs near 30%, and simulated studies showed its potential. For instance, Z. Abbasi *et al.*^29^ revealed the layout of CSTO/KGeCl_3_/nPB achieved 29.30% PCE with other performance parameters (*V*_OC_ ≈ 0.815 V, *J*_SC_ ≈ 41.8 mA cm^−2^, FF ≈ 85.97%), while FTO/SnS_2_/KGeCl_3_/Cu_2_O/C configuration achieved 15.83% PCE (*V*_OC_ ≈ 0.545 V, *J*_SC_ ≈ 41.91 mA cm^−2^, FF ≈ 69.24%) by M. A. F. Siddique *et al.* in 2024.^26^ Based on these findings, we are attempting to analyze KGeCl_3_ as an absorber with four efficient ETLs (WS_2_, ZnSe, PC_60_BM, SnS_2_) and other layers like fluorine-doped tin oxide (FTO) and CFTS. With the addition of artificial intelligence (AI) in solar technology, a new way of performance acceleration is unlocked with different machine learning (ML) approaches.^44–46^ Recently, Liu *et al.* in 2026 have proposed a deep learning-guided screening framework that accelerates the discovery of high-efficiency donor–acceptor pairs in organic photovoltaics by accurately capturing structure–property relationships. This technique is named SolarPCE-Net, and the synergistic interactions of the method demonstrate the growing impact of advanced AI architectures in PV materials discovery.^47^ This category of studies takes into consideration the cell's internal and external parameters for the optimization process. To further enhance device performance, many similar studies have considered the optimization of absorber thickness, doping density like donor density (*N*_D_) and acceptor density (*N*_A_), defect density (*N*_t_), interface defects (IL1, IL2), active temperature (aided by post-deposition thermal annealing), work-function alignment, and resistive losses (series and shunt) to be examined both theoretically and practically.^27,48–50^

In our proposed numerical modeling, lead-free KGeCl_3_ has been utilized as an absorber layer with four different ETLs (ZnSe, WS_2_, PC_60_BM, SnS_2_) and CFTS as an HTL layer. Those materials were selected for their excellent PV performance, high extraction capacity, and lower recombination as per previous studies. The KGeCl_3_ appears to have thermal and chemical stability, high PCE, proper mobility of electrons and holes, along with tunable bandgaps (1.1 eV), which increases the lifespan of the solar cell.^25,26,51^ Furthermore, we analyze the impact of different parameters such as thickness, *N*_D_, *N*_A_, *N*_t_, interface defect density (IL1 & IL2), temperature, work function, series and shunt resistance with the help of solar cell capacitance simulator (SCAPS-1D). Additionally, four supervised machine learning techniques, *i.e.*, random forest (RF), extreme gradient boosting (XGBoost), categorical boosting (CatBoost), and decision tree (DT), are symmetrically employed for the prediction of PCE and find out the important features for the performance enhancement. In a careful look, parameters like thickness, temperature, doping density, and series resistance highly affected their overall performance. The optimization is carefully considered to keep all material parameters in a realistic and practical range for future synthesis mechanisms. Furthermore, the sequentially optimized vital parameters consider high efficiency, low interface, and SRH recombination, and better stability.

## 2. Materials and methodology

### 2.1. KGeCl_3_-based PSC structure

**Fig. 1:**
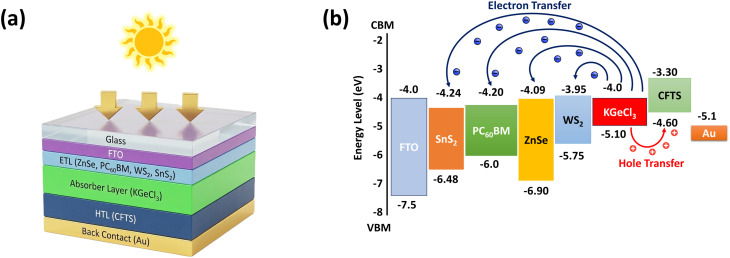
(a) Proposed perovskite structure for device performance simulation. (b) CBM and VBM position allocation of layers.

In this study, we have described four different topologies of perovskite solar cells with ZnSe, PC_60_BM, WS_2_, and SnS_2_-based structures. Fig. 1(a) shows a quick visualization of all four structures layer-wise. At each phase of the main development steps, we systematically adjusted our layer structures to guarantee the optimized material parameters to get enhanced device performance. The device configuration has several layers, with each layer contributing to device efficacy. The whole configuration consists of multiple layers, *e.g.*, FTO, ETL, absorber, HTL, and back contact. The structure begins with a glass substrate, followed by an FTO layer. This serves as the transparent conducting electrode. The ETL made of one of the four aforementioned materials is placed at the bottom of the FTO layer. The main absorber of KGeCl_3_ is capped by the ETL, whereas the HTL is made of CFTS. Additionally, the back contact layer is usually made of gold (Au), which improves charge collection and is used as another electrode for this structure. This architecture from Fig. 1(a) simultaneously shows how the active KGeCl_3_ layer absorbs photons and forms electron–hole pairs. These charges travel through the ETL and HTL layers, which serve as hole and electron blocking layers at the same time. Overall, the alignment of layers has a critical impact on the ultimate efficiency.

Fig. 1(b) depicts materials' energy levels alignment expressed in electron volts (eV). That contains two of the points, namely the conduction band minimum (CBM) and valence band maximum (VBM). The FTO layer functions as a transparent conducting electrode, has a CBM of −4.0 eV, and a VBM of −7.5 eV. The four CBMs of ETLs: ZnSe (−4.09 eV), PC_60_BM (−4.20 eV), WS_2_ (−3.95 eV), and SnS_2_ (−4.24 eV) have different band alignments with the other layers. Among these, WS_2_ is well aligned with the absorber layer KGeCl_3_, which has a CBM of −4.0 eV, eventually allowing for effective electron transmission from the absorber to the transport layer. The HTL, made up of CFTS, allows for hole transport at an energy level of −5.1 eV, while the back contact (Au) has a CBM of −5.1 eV, completing the hole transfer pathway. The improved device structure FTO/WS_2_/KGeCl_3_/CFTS/Au achieves a 19.22% initial PCE by slightly increasing the materials' original thermodynamic limits. Many new approaches have been illuminated by prior research; in conjunction with optimization to minimize the absorbent *N*_t_. These improvements were made *via* a wide number of methods, including changes to each layer's thickness and *N*_t_, as well as the density of donor and acceptor of layers.^52,53^ Furthermore, the effects of series-shunt resistance and temperature are also accounted for experiencing the consequence in a comparison with practical cells.^54^Fig. 1(b) additionally visualizes the initial energy band alignment with electron–hole pairs moving from the absorber layer to the CTLs. The ETL or hole blocking layer and HTL or electron blocking layer serve to remove charge carriers more efficiently with solid evidence.^55,56^ This efficiency is dependent on the layer's construction quality, which ultimately results in dramatically improved performance compared to earlier iterations.^57^Table 1 provides complete initial data on layer parameters. Moreover, Table 2 shows the primary interfacial parameters between the absorber/ETL and HTL/absorber interface. The initial temperature is adjusted to 300 K. AM 1.5G is the fixed sun illumination, and the solar irradiance is 100 mW cm^−2^. Relative convergence error tolerance and mesh point per layer are set to <10^−6^ and 200, respectively, for this whole simulation. For the back contact portion, this study is initiated with gold (Au), having the work function of 5.1 eV. No parasitic resistance (series and shunt) is introduced primarily for all configurations. These are the preliminary ingredients for making a successful evaluation of PV performance for all four structures.

**Table 1:** Materials properties and the corresponding parameters for SCAPS-1D simulation

Material property	FTO	CFTS	KGeCl_3_	WS_2_	ZnSe	PC_60_BM	SnS_2_
Thickness (nm)	200	100	500	100	70	50	50
Bandgap, *E*_g_ (eV)	3.5	1.3	1.1	1.8	2.81	1.8	2.24
Electron affinity, *χ* (eV)	4	3.3	4	3.95	4.09	4.2	4.24
Relative dielectric permittivity, *ε*_r_	9	9	23.01	13.6	8.6	4	10.0
Conduction band effective density of states, *N*_C_ (cm^−3^)	2.2 × 10^18^	2.2 × 10^18^	6.81 × 10^18^	1 × 10^18^	2.2 × 10^18^	1 × 10^21^	2.2 × 10^17^
Valence band effective density of states, *N*_V_ (cm^−3^)	1.8 × 10^19^	1.8 × 10^19^	3.45 × 10^18^	2.4 × 10^19^	1.8 × 10^18^	2 × 10^20^	1.8 × 10^19^
Electron thermal velocity (cm s^−1^)	10^7^	10^7^	10^7^	10^7^	10^7^	10^7^	10^7^
Hole thermal velocity (cm s^−1^)	10^7^	10^7^	10^7^	10^7^	10^7^	10^7^	10^7^
Electron mobility, *µ*_n_ (cm^2^ V^−1^ s^−1^)	20	21.98	92.92	100	400	0.1	50
Hole mobility, µ_h_ (cm^2^ V^−1^ s^−1^)	10	21.98	68.59	100	110	0.1	50
Donor density, *N*_D_ (cm^−3^)	10^18^	0	0	10^18^	10^18^	10^17^	10^17^
Acceptor density, *N*_A_ (cm^−3^)	0	10^18^	10^15^	0	0	0	0
Total density, *N*_t_ (cm^−3^)	10^15^	10^15^	10^15^	10^15^	10^15^	10^15^	10^14^
References	31 and 49	58	26 and 31	59	49	49	49 and 60

**Table 2:** Initial parameters of absorber/ETL (IL1) and HTL/absorber (IL2) interface defect layers^3,61^

Parameter	Properties	
	Absorber/ETL (IL1)	HTL/absorber (IL2)
Defect type	Neutral	Neutral
Electron capture cross section (cm^2^)	10^−19^	10^−19^
Hole capture cross section (cm^2^)	10^−19^	10^−19^
Energetic distribution	Single	Single
Energy level relative to *E*_v_	0.600	0.600
Total density (cm^−2^)	10^10^	10^10^

### 2.2. Energy band diagram

The conduction band offset (CBO) and valence band offset (VBO) pose essential characteristics in PSCs by consequently influencing charge carrier mobility and interface recombination.^43,62^ The KGeCl_3_ perovskite absorber layer (PAL) produces electrons and holes when stimulated in the structures in the SCAPS-1D simulation. Electrons must migrate from the PAL to the ETL, and holes should transfer from the PAL to the HTL. The dynamics of charge carriers are directly influenced by the CBO and VBO factors and the efficiency of charge extraction.^50^ To optimize and enhance carrier transport, the charge blocking situation between the HTL, PAL, and ETL should be eliminated through promoting seamless carrier transit. This diminishes energy barriers and decreases recombination. Conversely, the VBO at the PAL/HTL interface must be optimized to limit electron mobility and, at the same time facilitating effective hole transport. Correct alignment of the CBO and VBO at the layer interface guarantees optimal charge extraction with the lowest amount of recombination.^63^

**Fig. 2:**
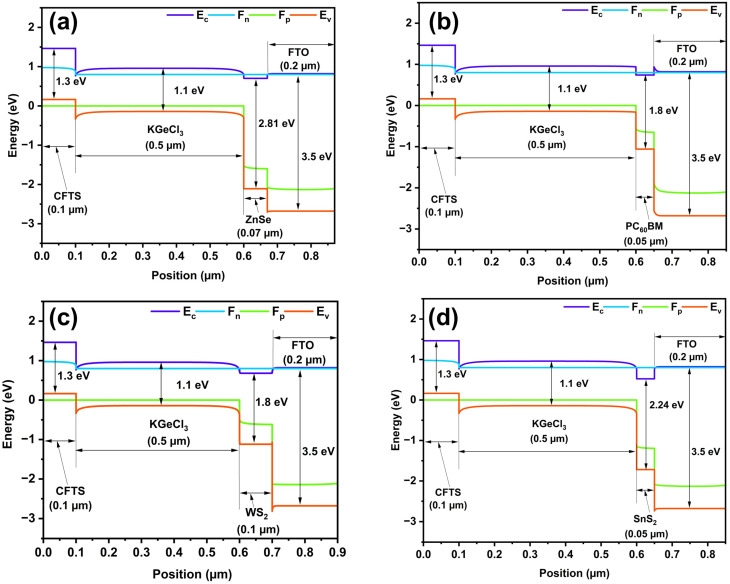
Energy band diagram of KGeCl_3_-based PSC with CFTS as HTL, and (a) ZnSe, (b) PC_60_BM, (c) WS_2_, and (d) SnS_2_ as ETL.

Fig. 2 depicts the band alignment of four distinct ETL materials in company with the absorber and HTL. Among the ETL materials, WS_2_ exhibits a small positive CBO of 0.05 eV with a shallow spike-type alignment. This type of alignment facilitates efficient electron extraction by effectively suppressing interfacial charge recombination. This moderate barrier is favorable for balancing carrier transport and recombination control at the ETL/absorber interface.

In contrast, ZnSe, PC_60_BM, and SnS_2_ demonstrate the negative CBO of −0.09 eV, −0.20 eV, and −0.24 eV, respectively, with the cliff-type band alignments. This negative offset promotes rapid electron transfer from the absorber to the ETL. However, they may also increase the interfacial recombination due to the absence of an energetic barrier. Therefore, appropriate optimization and passivation strategies are essential to mitigate surface recombination losses in these configurations.^49,64^ Conversely, both SnS_2_ and ZnSe exhibit larger positive VBOs of 1.80 eV and 1.38 eV, respectively. These values of VBO strongly block hole transport towards the ETL and help to reduce the leakage and recombination. PC_60_BM presents a moderate VBO of 0.90 eV, offering sufficient hole blocking capability and enhanced electron characteristics. Additionally, these ETLS are chemically stable and comparatively less toxic for sustainable cell making.^65^ For the choice of HTL, CFTS exhibits a VBO of −0.5 eV for hole transport with negligible recombination in HTL materials. CFTS serves as an efficient electron blocking layer, and, as evident from past surveys, it is an appropriate selection for HTL applications in PSCs.^66^ The congruence of CBO and VBO values among all layers is essential for reducing the parasitic resistances and enhancing charge carrier efficiency. Meticulous selection and engineering of these materials can yield highly efficient, stable, and durable PSCs (Table 3).

**Table 3:** Band offset values and types between the perovskite absorber and CTLs

CTLs	CBO (eV)	CBO type	VBO (eV)	VBO type
WS_2_	0.05	Spike	0.65	Spike
ZnSe	−0.09	Cliff	1.80	Spike
PC_60_BM	−0.20	Cliff	0.90	Spike
SnS_2_	−0.24	Cliff	1.38	Spike
CFTS	0.70	Electron barrier	−0.50	Cliff

### 2.3. SCAPS-1D methodology

The simulation is performed using SCAPS-1D software (version 3.3.12), and it was developed by Professor M. Burgelman at the University of Ghent.^67^ It is broadly used for simulating different solar cell configurations. In this study, SCAPS-1D software simulates our desired perovskite solar cell structure FTO/ETL/KGeCl_3_/CFTS/Au. The Poisson equation and continuity equation are the major elementary equations of SCAPS-1D. Poisson's equation derives the relationship between the electrostatic potential and the electron and hole distribution in a PSC. The mathematical expression for Poisson's equation is described through eqn (1)^31,68,69^1
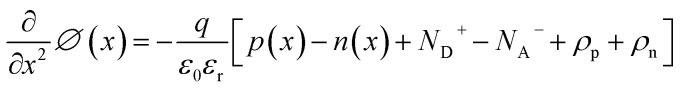
Here, *∅*(*x*) and *q* are the electrostatic potential and elementary electron charge, *ε*_r_ and *ε*_0_ is the relative permittivity and permittivity of free space, *p*(*x*) and *n*(*x*) hole and electron concentrations (cm^−3^), *N*_D_^*+*^ and *N*_A_^*−*^ is ionized donor and acceptor densities (cm^−3^), *ρ*_p_ and *ρ*_n_ is the hole and electron distribution.

Another important equation for the SCAPS-1D program is the continuity equation, which effectively calculates the rate of change of hole and electron carrier concentrations over position and time. The continuity equation describes how electrons or holes are generated, SRH recombined, or transported over time and space. It shortly expresses that the rate of change of carriers is equal to the net current flow + carrier generation – carrier recombination. The continuity equation can be described below with eqn (2) and (3):

Electron continuity equation2
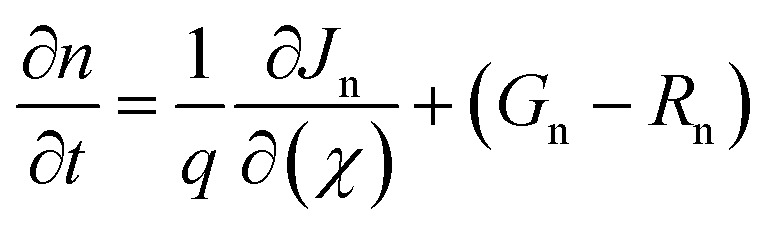


Hole continuity equation3
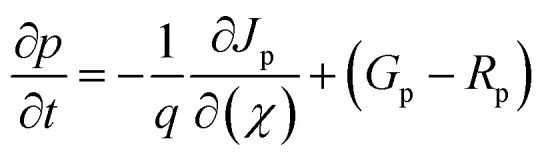
where, *J*_n_ and *J*_p_ stands for the current densities of electrons and holes. *G*_n_ and *G*_p_ depict the generation rate of electrons and holes. Lastly, *R*_n_ and *R*_p_ represent the recombination of holes and electrons.

Another important term for the simulation program is the carriers' drift-diffusion determination. Drift-diffusion characteristics are responsible for various factors, *e.g.*, efficient charge collection, generation-recombination mechanism, interface, and ionic effects. Those properties are also a key indicator for the selection and choice of effective layers for any solar cell. The charge carrier drift–diffusion equation for electrons and holes can be expressed by eqn (4) and (5)

4 *J*_n_ = *qµ*_n_*nε* + *qD*_n_∂*n*

5 *J*_p_ = *qµ*_p_*pε* + *qD*_p_∂*p*

where, *µ*_p_, *µ*_n_, *D*_n_, and *D*_p_ denotes the mobility of holes, mobility of electrons, diffusion coefficient of electrons, and diffusion coefficient of holes, respectively.

The performance parameters are the key factor to determine the overall effectiveness of any cell. For the evaluation of the parameters, the corresponding four parameters, including *V*_OC_, *J*_SC_, FF, and PCE has been maintained using the equations from (6)–(9)^31,69,70^6
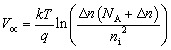
7
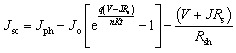
8
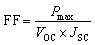
9
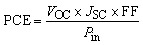
Here, the light-generated current and reverse saturation current are represented with *J*_ph_, and *J*_o_. Also, Δ*n* is the excess carrier concentration, and *k* is the Boltzmann constant. Moreover, *R*_s_ is a series resistance, *R*_sh_ is a shunt resistance, and *V*, and *J* represent the applied voltage and current density.

For the absorption profile of the incident photon, the sqrt of *E*_g_-model is chosen from the SCAPS-1D panel. This mathematical relation between the parameters for the absorption modeling can be expressed by the ‘Tauc law’ through eqn (10)–(12)10
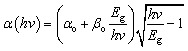
11
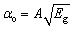
12
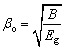
Here, *α*, *E*_g_, *hv* represents the coefficient of absorption profile, bandgap, and photon energy, sequentially. Additionally, *α*_o_ and *β*_o_ stands for the model parameters.

### 2.4. Machine learning algorithms

Machine learning (ML) can play a critical role in improving cell performance with high precision. For advanced cell manufacturing, ML has added a new dimension by using its predictive mechanism.^71,72^ Four supervised machine learning algorithms have been deployed to evaluate and predict the performance *via* a generated dataset of the SCAPS-1D package. The quick description of all four ML algorithms used in this study is given in the sections below:

#### 2.4.1. Random forest

Random forest (RF) is a supervised non-parametric learning technique that combines both classification and regression techniques. It has built up in combination with the prediction of different trees that may reduce the overfitting. As an ensemble learning technique, RF constructs a magnitude of uncorrelated decision trees during the training phase by utilizing bootstrap aggregating and random feature selection to make the model more dynamic. This ML technique is superior for its robust functionality against noisy data for further enhancement of the prediction accuracy.^73^ The mathematical approach for this ML algorithm can be described as eqn (13).13
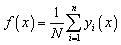
where, *f*(*x*) is the ultimate prediction and *y*_*i*_(*x*) is the *i*^th^ prediction, and *n* is the number of trees.

#### 2.4.2. Extreme gradient boosting

Extreme gradient boosting (XGBoost) is a powerful ensemble learning algorithm based on the gradient boosting framework, where multiple weak learners are sequentially combined to construct a strong predictive model. In XGBoost, decision trees are added iteratively, and each new tree is trained to minimize the residual errors produced by the previous ensemble. A distinctive feature of XGBoost is the regularization techniques to reduce the model complexity for significantly reduced data overfitting and enhance generalization performance. The algorithm incorporates both first and second order gradient information of the loss function while enabling faster convergence and highly accurate optimization in comparison with traditional gradient boosting methods. Due to its ability to capture non-linear relationships with input parameters, XGBoost is particularly suitable for modeling highly non-linear systems like PV devices.^74^ The model can be represented mathematically through eqn (14)14
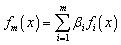
where, *f*_*m*_(*x*), *β*_*i*_, and *f*_*i*_(*x*) is the cumulative prediction, assigned weight of the *i*^th^ learner, and weak learning prediction of the *i*^th^ learner, sequentially.

#### 2.4.3. Categorical boosting

Categorical boosting (CatBoost) is a gradient boosting algorithm specifically developed to address the limitations of traditional methods to efficiently handle categorical and heterogeneous data. CatBoost employs an ordered boosting strategy that prevents target leakage and reduces prediction bias during the training procedure. This method ensures that the model learns from past observations, which makes this algorithm more stable and reliable. Furthermore, CatBoost automatically converts categorical variables using target-based statistical encoding with appropriate regularization. This phenomenon improves the learning efficiency without further preprocessing of the features. The training mechanism and reduced sensitivity to hyperparameter tuning make this model robust for regression-based performance prediction in solar cell optimization.^75^ The mathematical overview can be expressed as eqn (15).15
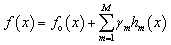
where, *f*_o_(*x*), *γ*_*m*_, *h*_*m*_(*x*) is the initial predicted value, the learning rate for *m*^th^ iteration, and *m*^th^ weak learner trained on the residues of the previous step, respectively.

#### 2.4.4. Decision tree

A decision tree (DT) is a non-parametric supervised learning algorithm that predicts outcomes by recursively partitioning the input features into smaller homogeneous regions. At each decision node, the algorithm picks the feature and corresponding threshold that effectively separates the data according to a predefined impurity criterion, which creates a tree-like hierarchical structure. This process continues until a stopping condition is met and predictions are obtained from the terminal leaf nodes. DTs are highly interpretable because of the underlying data structure and physical relationship between input and output responses. However, the overfitting tendency has arisen without constraints when the appropriate control of tree depth and minimum sample size is not carefully chosen.^76^ The mathematics behind this algorithm can be written as eqn (16)16
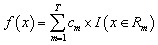
where, *T*, *c*_*m*_, and *R*_*m*_ is the total number of tree nodes, prediction values for *m*^th^, and *m*^th^ disjoint region (leaf) of the feature space, accordingly.

## 3. Result and discussion

### 3.1. Thickness optimization of ETL and HTL

The thickness of CTLs is essential for improving the PCE of perovskite solar cells. To serve this purpose, a range of thickness boundaries is chosen to figure out the performance of the simulated structures. A systematic variation between 20 and 200 nm was examined to figure out the best thickness range for CTLs.^77^ The optimal thickness of ETL and HTL is crucial due to their elevated charge carrier mobility, diminished recombination losses, and effective charge extraction.^78^ The optimization of these layers enhances not only the efficiency of PSCs but also affects stability and charge transport dynamics.^57^ Consequently, meticulous regulation of energy bandgap alignment in CTL materials is imperative.

**Fig. 3:**
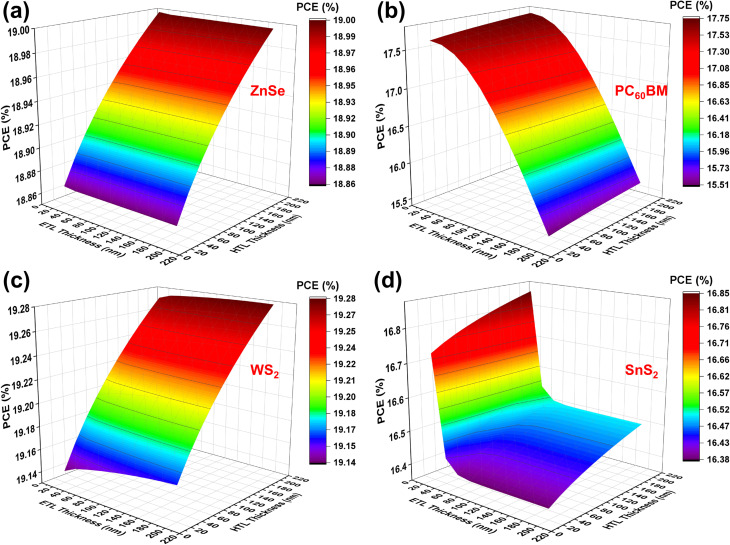
CTLs thickness variation effect on PCE (a) ZnSe, (b) PC_60_BM, (c) WS_2_, and (d) SnS_2_.

Fig. 3 portrays the variation impact of both ETL and HTL thicknesses to examine the ultimate PCE. According to the illustration of Fig. 3(a)-(c), ZnSe and WS_2_ ETLs exhibit a constant performance across the full thickness variation spectrum. This suggests that carrier extraction and recombination in these materials exhibit reduced sensitivity to the variations in ETL thickness. The configuraion of PC_60_BM exhibits lower thickenss sensitivity compared to the other configurations, as shown in Fig. 3(b). Conversely, SnS_2_ ETL has demonstrated a progressive decline in performance with increased thickness from Fig. 3(d). This is a major indication of elevated trap densities, diminished carrier mobilities, and augmented recombination losses.^56^ Among the ETL candidates, WS_2_ has exhibited the highest PCE of 19.21%, followed by ZnSe at 18.94%, PC_60_BM at 17.69%, and SnS_2_ at 16.80%, respectively. The outstanding performance of WS_2_ is proven for its bandgap of 1.8 eV and advantageous band alignment with PAL for enhanced and effective electron extraction. WS_2_ consists of tungsten atoms interspersed between hexagonally organized sulfur atoms, facilitating improved light absorption and charge transfer.^31^ In contrast, SnS_2_ and PC_60_BM have greater negative CBO values, which can result in an upshot of electrons at the ETL/absorber interface and diminished device performance. The best ETL thicknesses identified were 20 nm for WS_2_, ZnSe, PC_60_BM, and SnS_2_. For HTL CFTS, the PCE is rather stable across the same thickness variation range. This trend stands out as a major indicator of the negligible effect of HTL material thickness on hole transport and the generation, while improving charge collection by diminishing recombination.^50^ At an HTL thickness of 100 nm, similar peak efficiencies have been observed for ZnSe, WS_2_, PC_60_BM, and SnS_2_ ETLs.

### 3.2. Optimization of absorber thickness

**Fig. 4:**
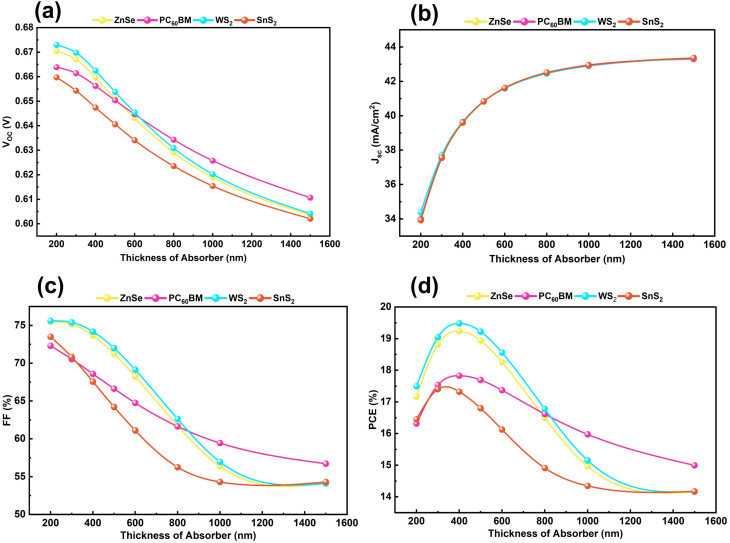
Alternation effect of absorber thickness on (a) *V*_OC_, (b) *J*_SC_, (c) FF, and (d) PCE, with four ETLs.

Fig. 4 portrays the effect of absorber thickness variation in the simulation from 200 to 1500 nm. Firstly, the ETL and HTL thicknesses have been set to their optimized values, and then the optimal thickness of the absorber layer has been investigated in this section. The absorber layer (KGeCl_3_) is particularly crucial to mark the cruciality of effectively PSCs perform since it directly impacts the amount of light the photon absorbance, carriers' generation and recombination, electric field generation, and carrier spread out mechanisms.^79^ This layer's ability to effectively absorb incoming photons makes an electron hole pair (EHP). Moreover, the material internal parameters depend a lot on the perovskite material's natural properties, especially its bandgap.^80^ To produce an effective layer for absorbing light, it needs to stop recombination and make carriers last longer with a prolonged carrier lifetime. Making the absorber thicker improves both the built-in electric field and the diffusion length. These things work together to make it easier for the charge carrier to move.^81^

From the beginning, Fig. 4(a) shows a decline pattern in *V*_OC_ due to the increased recombination rate for all four structures. As the absorber gets thicker, the short-circuit current density (*J*_SC_) rises quickly, as shown in Fig. 4(b). This is because the light is absorbed better, and the charge carriers are gathered more effectively. The larger *J*_SC_ signifies that more electrons and holes are being created, so the carrier concentration is higher, and the current output is stronger.^57^ Both Fig. 4(a) and (c) show that with the thickness increasing, the *V*_OC_ and FF are descending slowly. This pattern indicates increased recombination losses and falling charge extraction efficiency. Fig. 4(d) is the PCE curve with an observable peak at a 400 nm thick absorber, and thereafter slowly falling with WS_2_, ZnSe, and PC_60_BM after their optimum. While a thicker absorber increases photon absorption, it simultaneously increases the series resistance and the probability of carrier recombination before reaching the contacts.^81^ Thus, 400 nm represents the optimal balance between generation and collection efficiency. However, SnS_2_ configuration is best at a 300 nm layer thickness with its performance metrics. One important point to be noted, when the absorber is very thin, recombination is dominant because there are few carriers around to absorb incoming light. If we make it too thick, less charge can be produced from light because there's not enough surface area for photons to interact with our material.^53^ Therefore, the correct thickness allows most light to create charge whilst losing the least to defects. Finally, Fig. 4(d) demonstrates that at their respective best absorber thicknesses, different ETL materials can achieve the highest efficiencies of 19.46% for WS_2_, 19.24% for ZnSe, 17.83% for PC_60_BM, and 17.40% for SnS_2_. This study finds that 400 nm is the best thickness of KGeCl_3_ absorber for WS_2_, ZnSe, and PC_60_BM, but 300 nm is the optimum one for SnS_2_ structure.

### 3.3. Optimization of ETL donor density

**Fig. 5:**
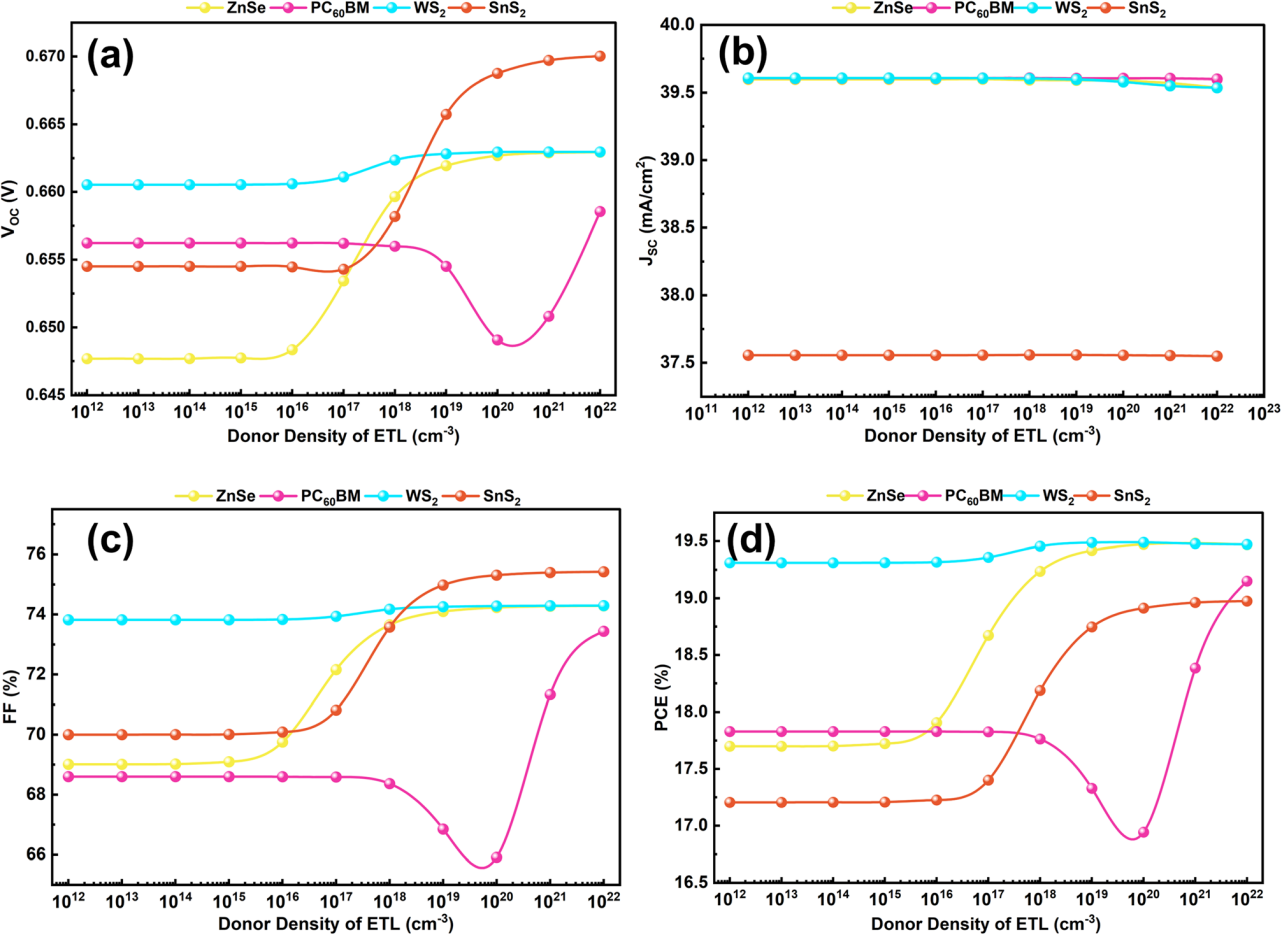
Performance parameters with the variation of *N*_D_ of ETL (a) *V*_OC_, (b) *J*_SC_, (c) FF, and (d) PCE.

In PSCs, donor density (*N*_D_) is an important term to determine free electron conductivity, charge separation efficiency, recombination losses, optical characteristics, and finally the overall performance of the device.^67^ A boost in donor density means the appearance of additional free electrons, which will change the electronic configuration and performance characteristics of PSCs. In this study, we try to investigate the optimal donor densities of different electron transport layers (ETLs). The initial defect densities of the ETLs are shown in Table 1, where 10^18^ cm^−3^ for WS_2_ and ZnSe, 10^17^ cm^−3^ for PC_60_BM, and SnS_2_. Fig. 5 plots the change in donor densities from the range of 10^12^ cm^−3^ to 10^22^ cm^−3^. As can be seen from Fig. 5(a), the *V*_OC_ of ZnSe, WS_2_, and SnS_2_ remains almost constant for a long time to 10^16^ cm^−3^. Beyond this, it begins to increase till the rest of the portion. In contrast, PC_60_BM maintains an unvarying *V*_OC_ up to 10^18^ cm^−3^, then suddenly drops off at 10^20^ cm^−3^ before climbing back up by 10^22^ cm^−3^. The increase in *V*_OC_ indicates a proper band alignment and less recombination at high donor densities. Then, in Fig. 5(b), the *J*_SC_ for all ETLs remains nearly the same within the variation range. This is an indicator that carrier generation and absorption of light by the ETL are not largely affected by variation in *N*_D_. Likewise, as shown in Fig. 5(c), the FF has a similar trend to *V*_OC_, with all ETLs showing a considerable improvement at higher donor concentrations. A high FF, which denotes good conductivity and rapid electron transport within the ETL, clearly impacts the PCE of all structures which as visualized in Fig. 5(d). For ZnSe, SnS_2_, and WS_2_, the PCE stays constant up to 10^16^ cm^−3^, then begins to rise after that. As for PC_60_BM, PCE remains level until 10^18^ cm^−3^, from which point it makes a sudden leap up at 10^22^ cm^−3^. The optimization of the *N*_D_ produced dynamic results; it achieved a maximum PCE of 19.49% with WS_2_, 19.47% with ZnSe, 18.91% with SnS_2_, and 17.83% with PC_60_BM configuration. The accompanied increase of *V*_OC_ and FF, together with almost unchanging *J*_SC_, combine to produce significant gains in device efficiency. Through optimization, charge extraction is improved, series resistance is reduced, and band alignment becomes sharper. These enhancements are thought to be the reason for increased capacitance and lengthened carrier lifetime. Furthermore, the optimal ETL donor densities were determined to be 10^20^ cm^−3^ for WS_2_, ZnSe, and SnS_2_, as well as 10^17^ cm^−3^ for PC_60_BM. Among the structures examined, the FTO/WS_2_/KGeCl_3_/CFTS structure overall gave the best performance.

### 3.4. Optimization of HTL acceptor density

**Fig. 6:**
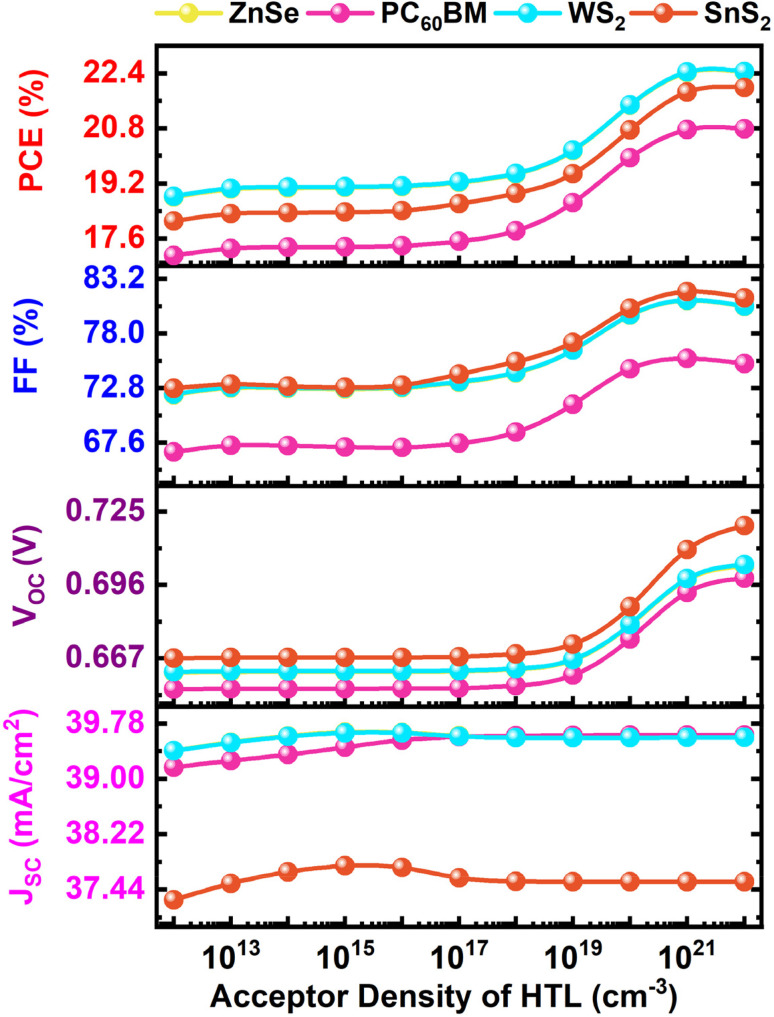
Acceptor density variation impact of HTL on performance parameters.

The acceptor density (*N*_A_) of the HTL is very important for controlling carrier transport, interfacial recombination, and the overall PCE. CFTS is a comparatively stable and low-cost material with better optical and electrical performance.^82^ When the HTL acceptor density goes from 10^13^ to 10^17^ cm^−3^, the PV parameters don't change much, as shown in Fig. 6. The *V*_OC_ exhibits low, the FF shows moderate, and the PCE is relatively poor in the variation region up to 10^18^ cm^−3^. In this low-doping regime, the weak band bending at the HTL/absorber interface does not create enough built-in potential for efficient charge separation. This makes it take longer to extract holes and increases recombination losses. The built-in electric field at the interface gets stronger when the doping density goes beyond 10^19^ cm^−3^. This speeds up carrier transport and slows down recombination at the heterojunction, which improves both *V*_OC_ and FF. In the high doping range of 10^21^ to 10^22^ cm^−3^, there is a gradual and sharp improvement in the performance parameters. The HTL makes a very conductive path for hole transport, lowers series resistance, and makes it easy to make almost ohmic contact at the interface. The PCE goes up a long way till 10^20^ cm^−3^, with the highest efficiencies being 21.47% for WS_2_, 21.46% for ZnSe, and 20.75% for SnS_2_, as well as 19.94% for PC_60_BM configurations. The *J*_SC_ stays almost the same across the whole doping range. This shows that HTL doping mostly affects electrical processes and not optical ones, and does not affect the creation of photocarriers. With previous studies, the *J*_SC_ curve stays virtually the same when the *N*_A_ of the absorber layer changes from 10^14^ to 10^18^ cm^−3^. However, when the doping level gets close to 10^22^ cm^−3^, the curve drops sharply because Auger and trap-assisted recombination start to happen.^83^ These recombination processes shorten the lifespan of carriers that eventually make the depletion region smaller and limit the diffusion length.^84^ This makes it harder for carriers to aggregate and lowers the photocurrent. This trend shows the importance of finding the right balance between doping concentration. A high HTL acceptor density is good for device performance, but too much absorber doping leads to a lowering of overall efficiency.

### 3.5. Impact of acceptor density variation of absorber

**Fig. 7:**
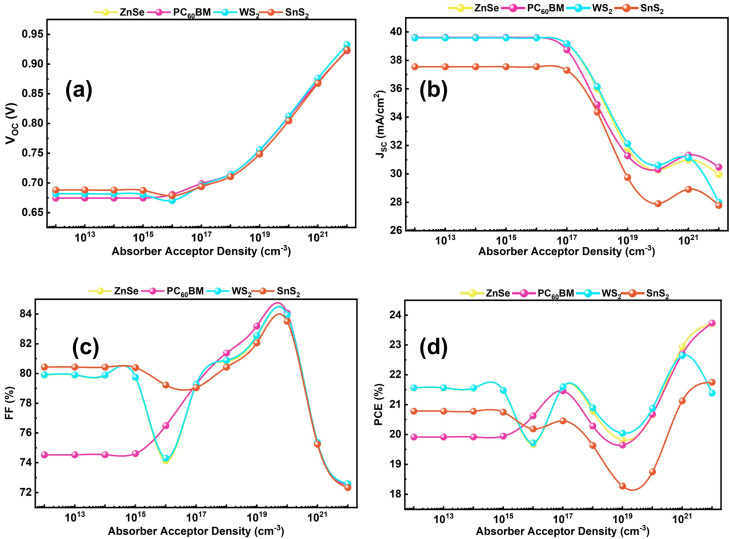
Variation impact of absorber *N*_A_ on cell performance (a) *V*_OC_, (b) *J*_SC_, (c) FF, and (d) PCE.

Initially, the absorber acceptor density was set to 10^15^ cm^−3^. The acceptor density (*N*_A_) varies from 10^12^ to 10^22^ cm^−3^. In Fig. 7(a), the *V*_OC_ remains constant at 10^14^ to 10^16^ cm^−3^; after that, it noticeably increases up to 10^22^ cm^−3^. The rising *V*_OC_ implies a strong build-up in the electric field, which improves the separation of carriers. Fig. 7(b) illustrates the *J*_SC_ curve, at 10^12^ to 10^17^ cm^−3^, the *J*_SC_ curve remains almost constant, then remarkably goes down at 10^22^ cm^−3^. The parameter *J*_SC_ has a reverse impact on the absorber layer, decreasing the short-circuit current with respect to higher *N*_A_, resulting in high Auger or trap-assisted recombination, a narrow depletion region, and reduced diffusion length.^58,68^ Again, Fig. 7(c) describes that the FF fluctuates up to 10^17^ cm^−3^, then significantly rises to the maximum point at 10^20^ cm^−3^. The rising trend of FF improved charge transport and lower series resistance. Lastly, the PCE trend for all configurations is described in Fig. 7(d). On a careful look, the *V*_OC_ and FF are increased; as a result, the overall performance is also improved. Furthermore, it is observable from the study that the increase beyond a threshold, absorber *N*_A_, depicts a bulk amount of recombination loss, high diffusion, and trap density state. However, an adequate amount of *N*_A_ appropriately affects device generation and recombination. Therefore, we have selected the optimized value of *N*_A_ at 10^17^ cm^−3^ for all ETL materials with the best performances for WS_2_, ZnSe, PC_60_BM, and SnS_2_ ETLs of 21.59%, 21.58%, 21.46% and 20.45%, respectively.

### 3.6. Defect density variation impact of ETL and HTL

**Fig. 8:**
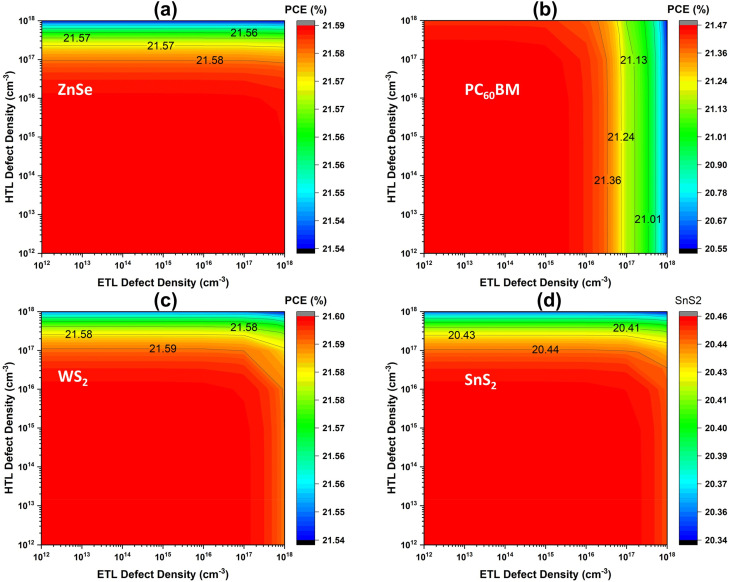
ETL and HTL's defect density variation impact on performance (a) ZnSe, (b) PC_60_BM, (c) WS_2_, and (d) SnS_2_.

Fig. 8 expresses the performance variation of CTLs with respect to their individual defect density. *N*_t_ is a supreme determinant due to the direct influence on charge separation and generation of PSC.^26,85^ Additionally, *N*_t_ has a direct influence on the generation, recombination, and carrier lifetime.^65^ Initially, the *N*_t_ of the ETL materials WS_2_, ZnSe, and PC_60_BM was considered to be 10^15^ cm^−3^, and SnS_2_ was assigned a *N*_t_ of 10^14^ cm^−3^. Similarly, the *N*_t_ of the HTL was set at 10^15^ cm^−3^. As shown in Fig. 8, the variations in ETL defect density and HTL defect density indicate that the photovoltaic parameters *V*_OC_, *J*_SC_, FF, and PCE remain nearly constant up to 10^16^ cm^−3^. Beyond this threshold, however, *J*_SC_ and PCE begin to decline, particularly in the case of WS_2_ and PC_60_BM materials. Moreover, it shows that variations in the HTL *N*_t_ display negligible influence on device performance, as the values of performance parameters remain almost unchanged across all four device structures. After optimizing *N*_t_ of ETL and HTL, the simulated PCEs for the four different structures were found to be 21.59%, 21.58%, 21.46%, and 20.45% for WS_2_, ZnSe, PC_60_BM, and SnS_2_, accordingly. These findings indicate that the ETL and HTL defect densities have a lower impact on the overall device performance. Therefore, we have marked the optimized ETL and HTL defect density as an initial value.

### 3.7. Effect of variation in absorber defect density

**Fig. 9:**
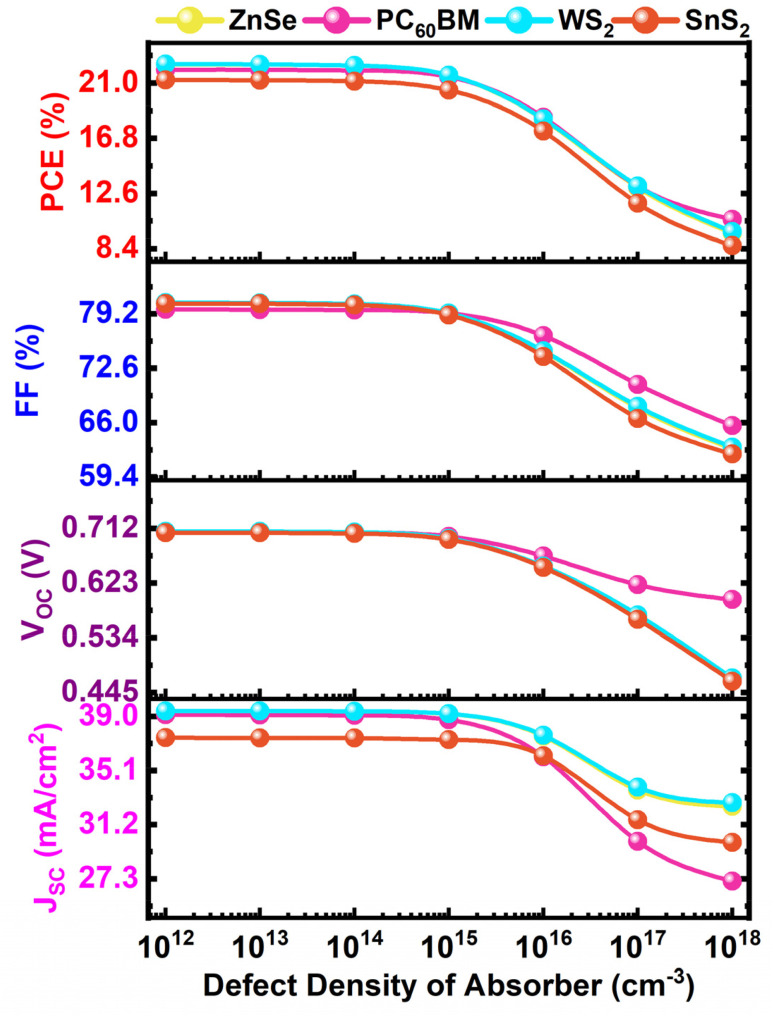
Variation effect of absorber defect density on cell performance.

The number of absorber defects is important for the impact on charge carriers' movement, recombination, and energy levels. This is a more dominating effect on performance than ETL and HTL.^78^ In Fig. 9, the device parameters show clear changes as the defect density goes up. When the defect density is modest in the range between 10^12^ to 10^14^ cm^−3^, *V*_OC_, FF, and PCE only go down a little, and *J*_SC_ stays almost the same. This stability in *J*_SC_ indicates that the photogeneration rate and carrier extraction are not substantially impacted at low trap densities *via* facilitating effective carrier collection. However, the defect density goes above 10^14^ cm^−3^, deep and shallow traps in the absorber start to take over. These traps operate as a recombination accelerator that speeds up non-radiative SRH recombination.^86^ The ongoing drop in *V*_OC_ is caused by more recombination losses for lowering the quasi-Fermi level splitting between electrons and holes as *N*_t_ goes up.^57^ This is an important factor in determining *V*_OC_. The drop in FF is also due to trap-assisted recombination and higher series resistance.^50^*J*_SC_ stays the same up to about 10^17^ cm^−3^, which means that photogeneration isn't significantly affected. But when defect concentrations are higher than this, recombination happens faster than carrier transport and drop photocurrent sharply. This change shows the internal electric field is weaker because of less drift-assisted carrier collection and boosts bulk recombination.^77^ The simultaneous decrease in PCE is the result of a combination of lower *V*_OC_, worse FF, and lower *J*_SC_. So, it's important to manage the *N*_t_ of the absorber in order to make the device work better. This work shows that the best and optimal defect density is 10^14^ cm^−3^, where recombination losses are low, and carrier transport is still efficient. In this case, the greatest PCE values for all ETLs are listed for WS_2_ for 22.33%, ZnSe for 22.32%, PC_60_BM for 21.95%, and SnS_2_ for 21.13%, respectively. This shows that reducing trap states in the absorber not only makes photovoltaic parameters more stable, but it also makes perovskite solar cells last longer and work better over time.

### 3.8. Optimization of interface defect density

**Fig. 10:**
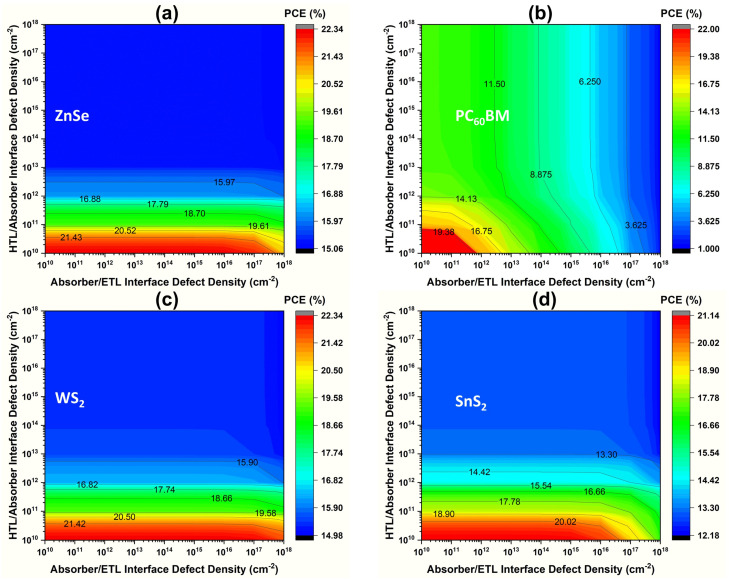
Variation impact on PCE of (a) ZnSe, (b) PC_60_BM, (c) WS_2_, and (d) SnS_2_ with interface defect density.

The defect density of the interface layer between the absorber/ETL layer (IL1) and the HTL/absorber layer (IL2) impacts the perovskite device performance, especially on the external quantum efficiency (QE).^87^ Hence, the necessity of optimization plays an inevitable role in enhancing the light absorption spectra while maintaining better stability and performance. In this study, Fig. 10 depicts the absorber/CTL total interface defect density with variations from 10^10^ to 10^18^ cm^−2^ for (a) ZnSe, (b) PC_60_BM, (c) WS_2_, and (d) SnS_2_ structures. The performance shown in these contour plots remains predominantly similar in nature across WS_2_, ZnSe, and SnS_2_ materials. The low-defect zone (below 10^13^ cm^−2^) has the highest efficiency, whereas deterioration is significant above 10^14^ cm^−2^. Better electronic coupling and lower non-radiative recombination losses are indicated by ZnSe and WS_2_, which also show improved interface tolerance and sustain PCE values above 20% throughout a larger defect range. On the other hand, even at relatively low defect densities, PC_60_BM exhibits a sharp decline in PCE, which indicates inadequate interface stability with the KGeCl_3_ absorber. In comparison to ZnSe and WS_2_, SnS_2_ exhibits moderate stability and a marginally lower peak efficiency. ZnSe and WS_2_ are the most attractive ETL choices for high-performance lead-free KGeCl_3_ perovskite solar cells. Overall, the results show that limiting interface defect concentrations, especially below 10^13^ cm^−2^, is crucial for obtaining optimal device efficiency. Therefore, we selected the optimized interface defect density of HTL/KGeCl_3_ and ETL/KGeCl_3_ at 10^10^ cm^−2^, based on the maximum performance. The best performance of the four different ETLs WS_2_, ZnSe, PC_60_BM, and SnS_2_ is 22.33%, 22.32%, 21.95%, and 21.13%, respectively.

### 3.9. Effect of back contact on performance

**Fig. 11:**
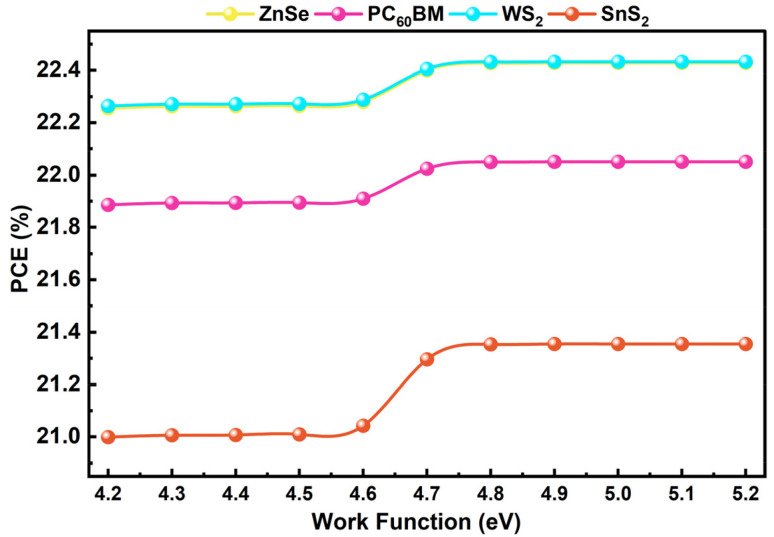
Work function alternation impact on PCE of different structures.

A good back contact is essential for a high-performing and stable PSC. It directly determines the charge extraction efficiency and the device's total lifespan.^88^ The back electrode serves not only as a physical layer but also plays a crucial role in determining the efficiency of carrier movement across the interface. Poor band alignment results in increased resistive losses, recombination, and diminished overall performance.^89^ In our study, we have tuned the back contact work function between 4.2 and 5.2 eV to see how it affects the PCE of PSCs with different ETLs (ZnSe, PC_60_BM, WS_2_, and SnS_2_). The visual representation of work function change in back contact can be found through Fig. 11. Primarily, due to poor band alignment and ineffective charge carrier extraction, the PCE slightly changes at the lower end (around 4.2–4.6 eV). However, there is a consistent improvement after the work function surpasses 4.6 eV, and the effect peaks between 4.8 and 5.2 eV. This is the area where the interface promotes freer charge movement, inhibits recombination, and reduces energy loss. At roughly 5.1 eV, which coincidentally corresponds to the work function of gold (Au), the highest performance is observed.^57^ This makes sense because Au forms an almost perfect energy level alignment with the primary absorber in addition to offering superb conductivity. At the same time, it minimizes losses while enabling effective hole collection towards the electrode. For this reason, Au is the ideal material for our study's final device structure, with the best performance coming from the constructed FTO/WS_2_/KGeCl_3_/CFTS/Au structure, with the recorded amount of PCE of 22.43%. This is one of the configurations that leads to long-term stability, easy carrier extraction, and maximum efficiency with this configuration.

### 3.10. Effect of parasitic resistance on cell performance

The performance of a PSC is strongly influenced by two key electrical factors, namely series resistance (*R*_s_) and shunt resistance (*R*_sh_).^3^ This parasitic resistance has a direct influence on the performance parameters. Conceptually, low *R*_s_ and high *R*_sh_ create the best conditions for stable and efficient operation. As *R*_s_ increases, the efficiency rapidly drops, regardless of *R*_sh_.^3^ This happens because higher series resistance limits current flow, introduces resistive losses, and distorts the current–voltage curve.^90^ On the other hand, *R*_sh_ decreases below 10^3^ Ω cm^2^ introduces leakage currents and improved recombination on the cell.^3^

**Fig. 12:**
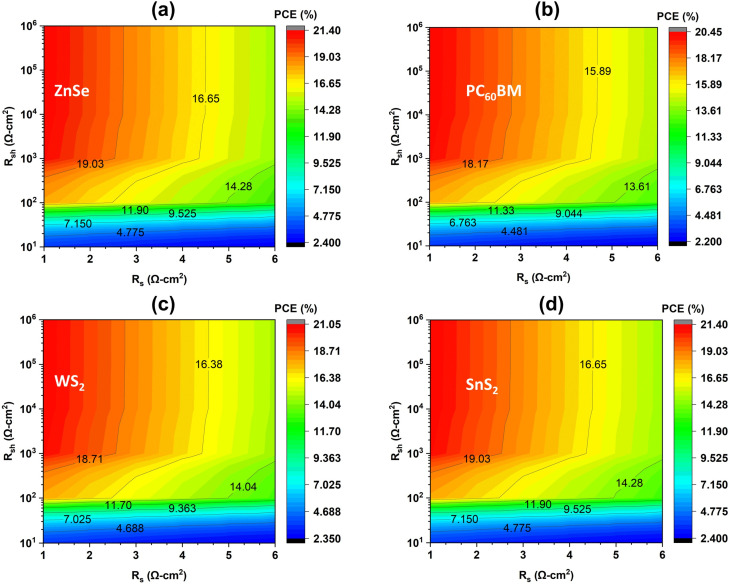
Contour plot of series resistance and shunt resistance variation on the PCE (a) ZnSe, (b) PC_60_BM, (c) WS_2_, and (d) SnS_2_.

Fig. 12 represents the variation effect of both parasitic resistances, *i.e.*, *R*_s_ and *R*_sh_. Among the four distinct ETLs, WS_2_ and ZnSe stand out for maintaining the highest PCE values even under moderately high *R*_sh_. This highlights their strong compatibility with the absorber. Another ETL PC_60_BM performs slightly lower but still shows a stable profile. Lastly, SnS_2_ gives the weakest performance among all the ETLs. This analysis reinforces that controlling both resistances is extremely important, besides optimizing the absorber and contacts. The optimal operating condition has been determined for low series resistance (<1 Ω cm^2^) with high shunt resistance (>10^4^–10^6^ Ω cm^2^) for extracting maximum power from the simulated devices. From this study, the best balance and optimization is achieved at *R*_s_ and *R*_sh_ of 1 Ω cm^2^ and 10^6^ Ω cm^2^, respectively. Under these optimized conditions, the highest efficiencies have been recorded as 21.39% for WS_2_, 21.38% for ZnSe, 21.05% for PC_60_BM, and 20.43% for SnS_2_, accordingly. These results clearly show the smooth charge transport, minimal losses, and reliable long-term performance of the device.^53,65^

### 3.11. Effect of operating temperature

The temperature also has a significant impact on PV performance and device stability. The increasing trend in device temperature leads to high recombination, which reduces the *V*_OC_ and causes material and interface degradation.^60,69,91^ For better understanding, the *V*_OC_, *J*_SC_, and *J*_o_'s relation with the temperature equations given in (17)17
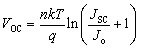


**Fig. 13:**
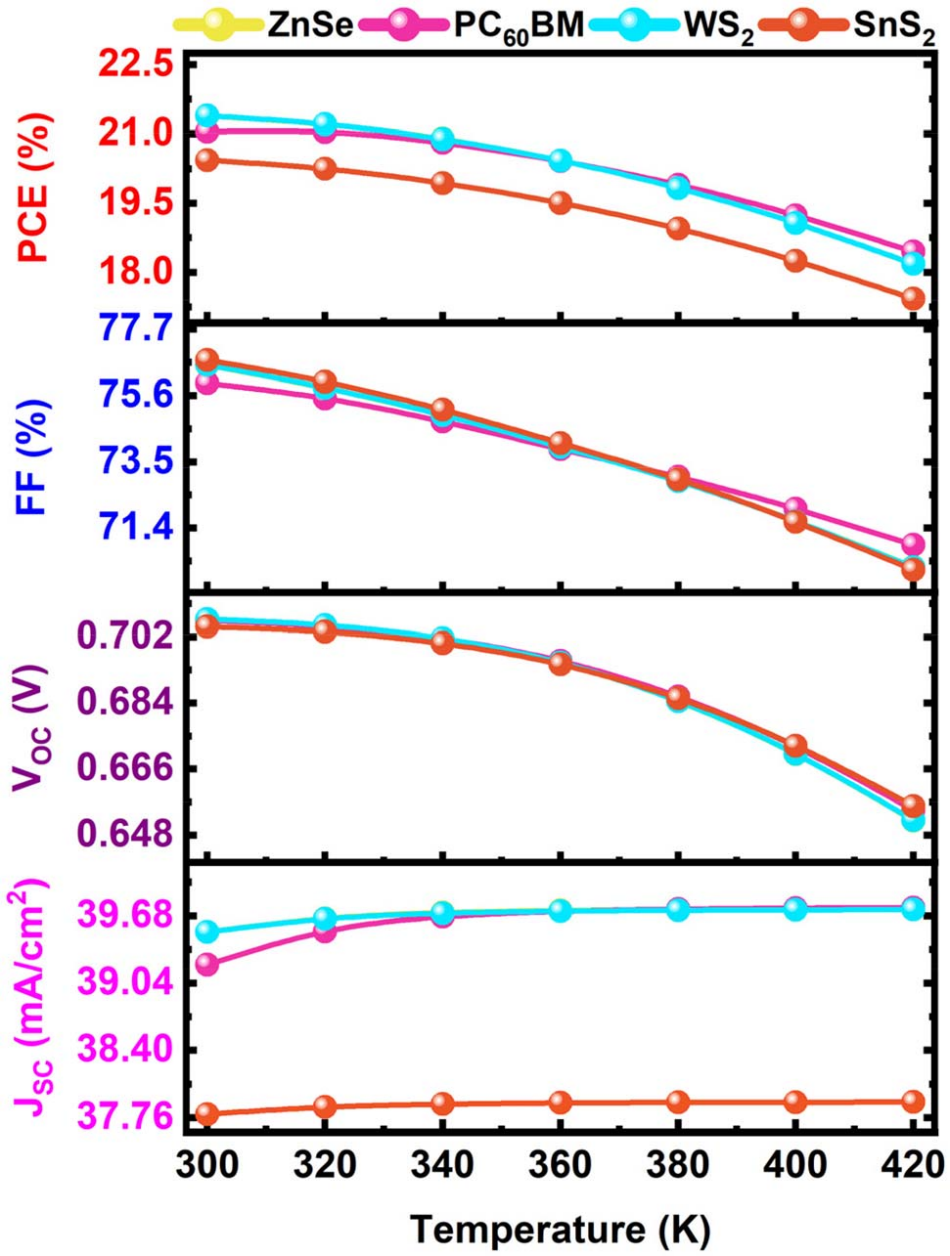
Effect of temperature on four different structures on cell parameters.

In Fig. 13, the temperature variation is taken from 300 to 420 K for comparison with the real environment. The *V*_OC_, FF, and PCE gradually decrease with rising temperature because of thermal instability, increasing series resistance, worsened charge collection, and increased leakage paths.^69^ When the temperature (*T*) increases, the thermal energy increases, and the dark saturation current (*J*_o_) increases exponentially due to enhanced intrinsic carrier concentration and recombination, resulting in *V*_OC_ gradually decreasing.^65^ Moreover, the FF and *V*_OC_ are proportional to the PCE due to decreased efficiency. But the *J*_SC_ remains constant because of the better carrier mobility, the photogeneration rate remains unchanged, and high light absorption. At a room temperature of 300 K, the best PCEs are obtained for four different ETL configurations of WS_2_, ZnSe, PC_60_BM, and SnS_2_, which are 21.39%, 21.38%, 21.05% and 20.43%, respectively.

### 3.12. Optimized *J*–*V* and QE curve

In this study, we optimized four different ETL configurations with WS_2_, ZnSe, PC_60_BM, and SnS_2_-based structures using a KGeCl_3_ absorber material and CFTS as hole transport material. The thickness of the ETLs and HTL has been varied in a range of 20 nm to 200 nm, while the thickness of the absorber material has been adjusted between 200 nm and 1500 nm. Additionally, the *N*_D_ of the ETL varies from 10^12^ to 10^22^ cm^−3^, and the *N*_A_ of both the absorber and HTL has also been analyzed over a range of 10^12^ to 10^22^ cm^−3^. The *N*_t_ of the absorber, HTL, and ETL materials was varied from 10^12^ to 10^18^ cm^−3^. Moreover, the interface defect densities at both the ETL/absorber and HTL/absorber junctions were adjusted from 10^10^ to 10^18^ cm^−2^. After final optimization, the PCE for the four distinct ETLs has been found to be as follows: 21.39% for WS_2_, 21.38% for ZnSe, 21.05% for PC_60_BM, and 20.43% for SnS_2_ accordingly. These results indicate that optimization significantly improved the PCE of all materials. On a careful look, it is proven that WS_2_ achieves the highest efficiency, closely followed by ZnSe. The enhancements in PCE result from optimized material characteristics, including decreased defect density, enhanced crystalline properties, and improved charge transport.

**Fig. 14:**
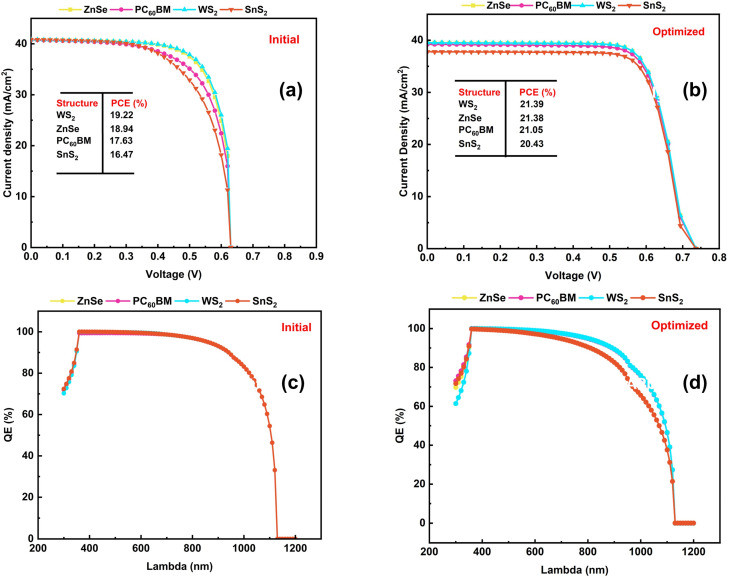
(a) Initial, and (b) optimized *J*–*V* curve, (c) initial, and (d) optimized QE for all four different structures.

Fig. 14(a) and (b) presents the *J*–*V* characteristics for the four ETL configurations. The trend of the illustration reveals the *J*_SC_ increment at lower values of *V*_OC_. This behavior emphasizes the balance between the generated *V*_OC_ and *J*_SC_ to achieve the maximum PCE in PV devices. The initial *J*–*V* structures illustrate the results derived from the simulation described in Fig. 14(a). It also shows the *J*–*V* characteristics of the ETL with the highest performance, WS_2_, which shows that it is still the best-performing ETL even in its initial state. The optimized *J*–*V* curves from Fig. 14(b) again show the highest PCE by WS_2_, which confirms its superior performance as an ETL material in this study. The curve shows that the device has a higher *V*_OC_ and *J*_SC_, which gives it the highest PCE of all the materials examined. This graph highlights that WS_2_ was successfully optimized, which led to a big increase in the efficiency of the device. Fig. 14(c) displays the improved devices' QE as a function of wavelength for the initial condition. The graph shows that the conversion of photons to electrons is quite good in the visible range, which is the section of the solar spectrum with the maximum energy. The device gets almost 100% QE from 400 nm to 700 nm of the incident photons. This means that the material is very good at turning photons into electrical charge in the visible range. This is significant for making solar cells work efficiently because the visible light spectrum makes up a large part of the total solar energy available. Fig. 14(d) shows the optimized QE for the best-performing technology. Here again, WS_2_ shows even more potential in the photon conversion to electrons. The graph simultaneously confirms that WS_2_ has a high QE over the visible spectrum and the ability to collect and transform light energy more efficiently. WS_2_ is remarked as the best ETL material in this investigation because it has a near-perfect QE spanning the 400 to 700 nm range. The KGeCl_3_ absorber, CFTS HTL, and the ETLs altogether have contributed a significant number to the elevated device outcome. These results remind the importance of optimizing material parameters like defect densities, thicknesses, and donor/acceptor concentrations to make an upgraded optoelectronic device for the next PV era.

### 3.13. Analysis of the generation and recombination rate

**Fig. 15:**
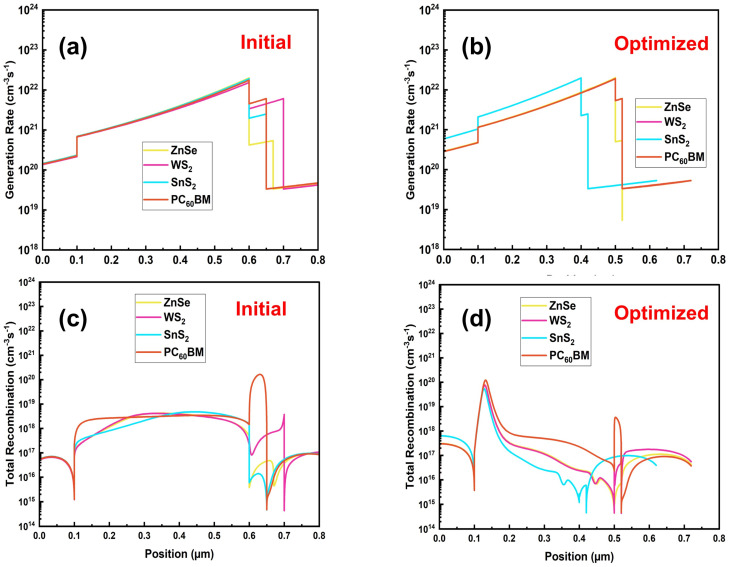
(a) Initial, (b) optimized generation rate, (c) initial, and (d) optimized total recombination rate.

Generation and recombination mechanisms are one of the core phenomena of solar cells. This is an utmost indicator to understand the generated carrier density ready to collect. In Fig. 15(a) and (b), the initial and optimized generation rate values for all four structures are illustrated. The optimized generation of all materials shows a smoother curve and increased overall performance. Also, it is suggested that a high magnitude, smoother distribution, and reduced recombination at the interface for the carrier. The overall generation rate across 0.1 to 0.6 µm shows its peak for all four structures. For mathematical calculation, the generation profile of carriers can be calculated by eqn (18),^69^

18 *G*(*λ*,*x*) = *α*(*λ*,*x*) × *N*_phot_(*λ*,*x*)

where, *G*(*λ*,*x*), *α*(*λ*,*x*), and *N*_phot_(*λ*,*x*) stands for generation profile, absorption profile, and incident photon flux, sequentially.

Fig. 15 also illustrates the overall position-dependent generation and recombination profiles of four distinct ETL configurations with multilayer solar cells under AM 1.5G light, contrasting the initial and optimized configurations. In the initial device structure from Fig. 15(a), the carrier production profile progressively increases across the absorber but displays significant spatial non-uniformity at the same time. This non-uniformity signifies an uneven optical field distribution and non-uniform photon absorption, resulting in enhanced interfacial recombination and inefficient carrier extraction. Conversely, the improved device from Fig. 15(b) exhibits a far smoother and robust generation profile with peak values nearing 2 × 10^22^ cm^−3^ s^−1^ with the optimized WS_2_-based structure. This enhancement results from structural modification, such as adjusting the thickness value of active layers, matching intermediate band alignment, or a higher optical absorption coefficient of layer materials.^86^ Those properties facilitate improved light trapping and capture of the incident photon spectrum from the sun. The recombination profiles with Fig. 15(c) further reinforce the impact of optimization. The initial structure has a recorded recombination peak of approximately 1.7 × 10^20^ cm^−3^ s^−1^ near the absorber/ETL interface. This finding indicates substantial interfacial defect-assisted recombination. This peak also signifies that charge carriers generated near the interface are rapidly dissipated before they can contribute to the photocurrent.^83^ In the post-optimization scenario of WS_2_ configuration, the recombination peak is markedly reduced to around 7.91 × 10^19^ cm^−3^ s^−1^, which is visible from Fig. 15(d). The occurrence of recombination indicates improved electronic quality of the junctions, perhaps due to better band alignment between the absorber and buffer layers, reduced defect density by interface passivation, or appropriate doping concentrations after optimization.^50,92^ The concurrent rise in carrier generation and decline in recombination indicates a comprehensive enhancement in the optical management and electrical quality of the device.^93^ These enhancements are expected to increase carrier collection efficiency, reduce losses, and thus improve PV performance metrics.^94^

### 3.14. Earlier studies comparison

Perovskite is a trendy research topic in this decade for its potential to create a breakthrough for the next generation of PV technology. To push up the ongoing enhancement, optimization is a must to meet requirements because of its necessity for making an efficient solar cell by eradicating all boundaries and constraints. Additionally, the process helps to make a cost-effective, durable, and reliable PSC in real life. In our study, we have conducted a full set optimization of absorber layer, ETL, and HTL parameters for maximizing the performance metrics. The optimization process has made a remarkable change in initial efficiency and ensures the stability of the simulated structures.

The study represents the outcome of KGeCl_3_-based PSC through SCAPS-1D simulation. Alike types of numerical studies had been conducted previously. But one of the major concerns of this study is the lack of experimental validation with the same materials in contemporary times. It is extremely rare to see the particular KGeCl_3_ perovskite absorber used for practical synthesis or manufacturing, to the author's best knowledge. But, Ge-based perovskite is a wide and potential weapon to replace the traditional lead-based perovskite, which is extremely hazardous both for human health and the environment.^28^ A few years back, in 2018, a comparatively nearby Ge-based structure of ITO/PEDOT:PSS/MAGeI_2.7_Br_0.3_/PC_70_BM/Ag reported a poor PCE of 0.57% due to the practical synthesis boundary for higher parasitic resistance.^95^ Other lead-free alternatives have been actively explored to address these concerns. Commonly, Sn-based absorbers such as CsSnI_3_ integrated into ITO/PCBM/CFTS/Se structures have delivered PCEs of 24.73% with *J*_SC_ values approaching 34 mA cm^−2^.^67^ Similarly, double perovskite systems such as Cs_2_PtI_6_ in an FTO/SnS_2_/Cs_2_PtI_6_/MoTe_2_/Au configuration reported PCE as high as 32.98%,^85^ though challenges in large-scale fabrication and stability persist. Germanium-based one can be a potential solution reported in prior studies. Against this background, KGeCl_3_ has recently emerged as a promising germanium-based perovskite alternative. Prior studies on KGeCl_3_ absorbers demonstrated efficiencies ranging from 15% to 29%, depending on device architecture.^25–27,29^ These results confirm that KGeCl_3_ can deliver competitive efficiencies comparable to or exceeding many lead-free systems.

The optimized device structure of FTO/CFTS/KGeCl_3_/WS_2_/Au has achieved the highest PCE of 21.39%, with a *V*_OC_ of 0.706 V, *J*_SC_ of 39.526 mA cm^−2^, and FF of 76.56% in this work. Other comparable efficiencies have been observed for ZnSe (21.38%), PC_60_BM (21.05%), and SnS_2_ (20.43%) as ETLs at the same time. A complete list of prior studies and our conducted study is highlighted in Table 4. Although these values are slightly lower than the highest reported KGeCl_3_ devices in literature, they remain significantly higher than several earlier experimental reports, for example, 15.83% for FTO/SnS_2_/KGeCl_3_/Cu_2_O/C.^26^ The strong *J*_SC_ values observed in this study (39–40 mA cm^−2^) indicate enhanced light absorption and efficient carrier collection due to careful selection and optimization of transport layers. KGeCl_3_ has competitive performance with the added benefit of non-toxicity and probable long-term stability, making it an appealing contender for sustainable solar applications, even if lead-based perovskites continue to have the greatest reported efficiencies. All things considered, the comparison shows that KGeCl_3_ is a feasible lead-free perovskite absorber that can achieve efficiencies higher than 21% in ideal simulated conditions.

**Table 4:** Comparative assessment of PV parameters for different KGeCl_3_-based and other reported solar cells

Type^a^	Device structure	*V*_OC_ (V)	*J*_SC_ (mA cm^−2^)	FF (%)	PCE (%)	Year	Ref.
E	FTO/SnS_2_/Cs_0.05_(FA_0.83_MA_0.17_)_0.95_Pb(I_0.83_Br_0.17_)_3_/Spiro-OMeTAD/Au	0.99	20.75	67.00	13.77	2020	36
E	ITO/PCBM/MAPbI_3_/PEDOT:PSS/Ag	0.971	19.04	46.54	8.60	2023	96
T	FTO/TiO_2_/KGeCl_3_/Spiro-OMeTAD/Au	1.02	25.77	78.25	22.98	2025	25
T	FTO/SnS_2_/Sr_3_PBr_3_/Sr_3_NCl_3_/Ni	1.27	26.44	90.14	30.32	2024	77
T	ITO/PCBM/CsSnI_3_/CFTS/Se	0.87	33.99	83.46	24.73	2023	67
T	FTO/SnS_2_/Cs_2_PtI_6_/MoTe_2_/Au	1.11	33.19	88.89	32.98	2024	85
T	ITO/WS_2_/KGeCl_3_/CBTS/Ni	0.679	41.439	78.12	22.01	2024	97
T	FTO/WS_2_/KGeCl_3_/PEDOT:PSS/Au	1.19	30.08	82.74	29.82	2024	98
T	FTO/SnS_2_/RbPbI_3_/MoO_3_/Ni	1.22	32.21	83.27	32.72	2025	99
T	FTO/WS_2_/KGeCl_3_/MoO_3_/Au	0.88	41.45	81.76	29.83	2024	31
T	FTO/SnS_2_/KGeCl_3_/Cu_2_O/C	0.545	41.91	69.24	15.83	2024	26
T	FTO/CSTO/KGeCl_3_/nPB/Au	0.815	41.804	85.97	29.30	2025	29
T	FTO/ZnSe/NaZn_0.07_Ag_0.3_Br_3_/ZnTe/Au	1.59	21.90	78.92	27.59	2024	94
T	FTO/SnS_2_/AgCdF_3_/C_6_TBTAPH_2_/Au	0.86	41.23	86.68	30.81	2026	100
T	FTO/WS_2_/KGeCl_3_/CFTS/Au	0.706	39.526	76.56	21.39	2025	This work
T	FTO/ZnSe/KGeCl_3_/CFTS/Au	0.706	39.525	76.55	21.38	2025	This work
T	FTO/PC_60_BM/KGeCl_3/_CFTS/Au	0.706	39.215	75.98	21.05	2025	This work
T	FTO/SnS_2_/KGeCl_3_/CFTS/Au	0.704	37.791	76.72	20.43	2025	This work

a E = experimental, T = theoretical.

The considerable variation in PV performance parameters has reported in Table 4 from fundamental differences in device architecture, material selection, and research methodology. The distinction between theoretical simulations and experimental studies is particularly significant. Previously reported simulations often predict higher efficiencies by assuming several factors, like ideal interfaces, optimal band alignment, and minimal defect densities. Conversely, experimental results must face the real-world challenges such as film non-uniformity, interfacial recombination, and material degradation.^4,10^ For example, the theoretical PCEs exceeding 29% for KGeCl_3_-based cells utilize CTLs like Spiro-OMeTAD, PEDOT:PSS, or MoO_3_.^31,98,101^ These types of materials provide excellent band alignment. But they are known to compromise long-term stability due to hygroscopic and thermal instability.^80,99,102^ In contrast, our study intentionally employs a fully inorganic stack like WS_2_ as the ETL and CFTS as the HTL. The choosing criterion prioritizes environmental resilience and thermal stability over absolute peak efficiency. The architectural choice for this study results in a slightly lower simulated PCE compared to some theoretical benchmarks, but the whole optimization has done with keep in mind to manufacturable pathway for practical device manufacturing. For our study, the WS_2_-based structure shows a shallow spike alignment (Δ*E*_c_ = +0.05 eV), which facilitates efficient electron extraction while suppressing interface recombination. On the other hand, SnS_2_'s cliff-type alignment can enhance interfacial losses if not properly passivated. Thus, our optimized efficiency of 21.39% with WS_2_ should be interpreted as a balanced achievement. Moreover, this architectural design focuses on respectable performance with enhanced durability and environmental compatibility for the next-generation PSC.

## 4. Machine learning integrated exploration

The machine learning (ML) analysis highlights a critical insight into the complex physics governing PSC performance. This previous observation strongly justifies the use of an advanced ensemble machine-learning model to capture higher-order interactions beyond the scope of conventional model simulation.^71,74,90^ This combined predictive–interpretive analysis demonstrates as a powerful surrogate for physics-based device analysis.

### 4.1. Correlation matrix analysis

**Fig. 16:**
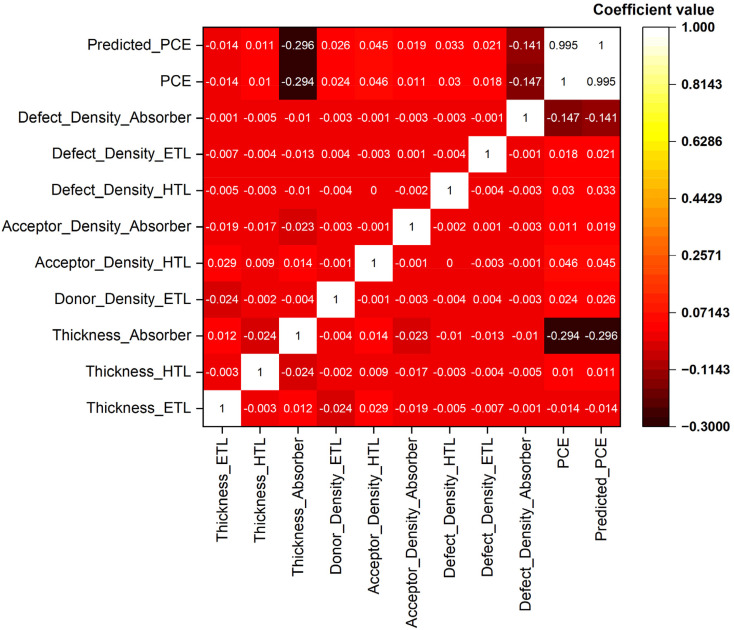
Pearson correlation matrix representation between different features.

The CTLs and absorber material's intrinsic parameters in from of a correlation matrix measured through the SCAPS-1D study and as anticipated by machine learning are shown in Fig. 16. This quantifies the linear correlations among the important device characteristics, where positive numbers imply direct linear relationships, whereas negative values indicate inverse linear dependencies. There is low multicollinearity in the dataset, as seen by the weak pairwise linear correlations among most input features in the correlation map. It is confirmed that the dataset covers a large and physically relevant parameter space free of artificial coupling by the near-zero off-diagonal values of thickness, doping densities, and defect densities. The absorber layer thickness has the strongest association with PCE (*r* = −0.294) out of all the input parameters, indicating a substantial inverse linear relationship. While a thicker absorber improves optical absorption, an overly thick one might make bulk recombination and carrier transport constraints worse, leading to decreased efficiency. This behavior is a result of a basic trend in semiconductor device physics. The relation with ML models has an edge over linear regression methods because they can capture this nontrivial equilibrium. It is consistent with the known role of trap-assisted Shockley–Read–Hall recombination in limiting carrier lifetime and quasi-Fermi level splitting. The effect of absorber *N*_t_ exhibits a clear negative correlation with PCE (*r* = −0.147). On the other hand, there is a considerable dependence on charge-selective contacts and interfacial energetics for the nonlinear impact of transport layer defect concentrations on device performance. They have shown almost nonlinear association with PCE.

Notably, the trained ML model confirms its extraordinary prediction accuracy and consistency by showing an almost perfect linear correlation (*r* = 0.995) between the expected and real PCE from SCAPS-1D. The fact that the model has learned physically relevant associations instead of false statistical artifacts is further supported by the near-identical correlation patterns of PCE and predicted PCE with respect to all input features.

### 4.2. Analysis of prediction *vs.* actual values for different algorithms

**Fig. 17:**
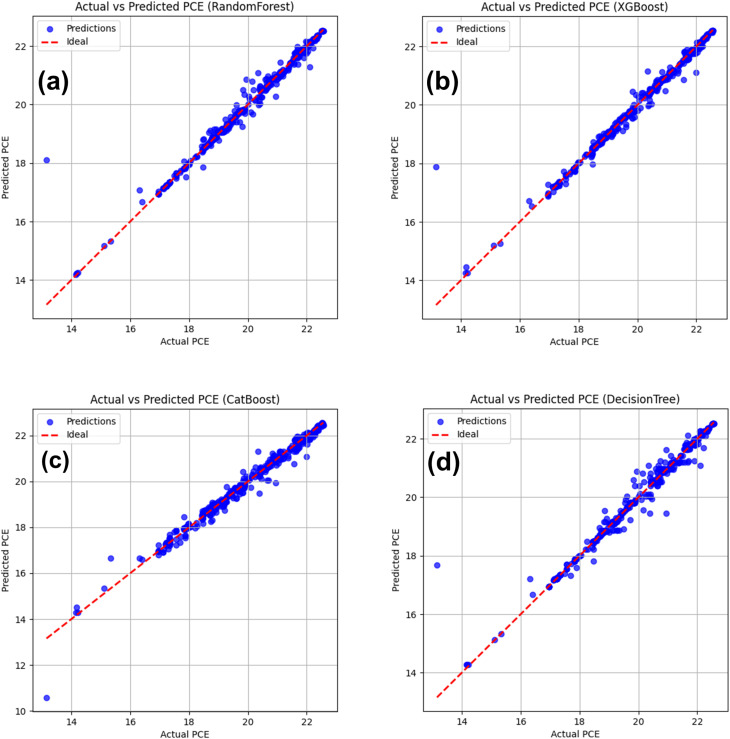
Comparison of actual *vs.* predicted PCE with four supervised ML algorithms (a) RF, (b) XGBoost, (c) CatBoost, and (d) DT.

Fig. 17 represents all four-machine learning (ML) algorithms that can reveal the relation between the actual and predicted PCE for all four structures. It presents the parity plots comparing calculated (actual) and machine-learning-predicted power conversion efficiency (PCE) values using four different regression models: (a) RF, (b) XGBoost, (c) CatBoost, and (d) DT. The reason behind choosing this model for their consistent performance for this kind of similar analysis from past.^71–74^ The RF model for Fig. 17(a) demonstrates a strong linear correlation between predicted and actual PCE values, while most data points are closely clustered around the ideal line. This incident indicates the event of high predictive reliability and minimal systematic bias. Again, a comparable trend is observed for the XGBoost model *via* slightly tighter clustering in the mid-to-high PCE range from Fig. 17(b). This algorithm potentially reflects the capability to capture nonlinear feature interactions to enhance the PSC performance.

Among all models, CatBoost exhibits the closest agreement with the ideal prediction line across the entire PCE range from Fig. 17(c). The algorithm CatBoost always suggests superior generalization and robustness against overfitting.^103^ In contrast, the DT model in Fig. 17(d) displays a relatively larger dispersion of data points at higher PCE values. This trend implies reduced predictive stability due to its inherent sensitivity to data partitioning. Overall, the parity analysis shows that ensemble learning techniques, particularly CatBoost and XGBoost, greatly outperform for this study. Those algorithms are demonstrating their efficacy for precise and trustworthy PCE prediction in PSC optimization.

### 4.3. SHAP interpretability analysis

**Fig. 18:**
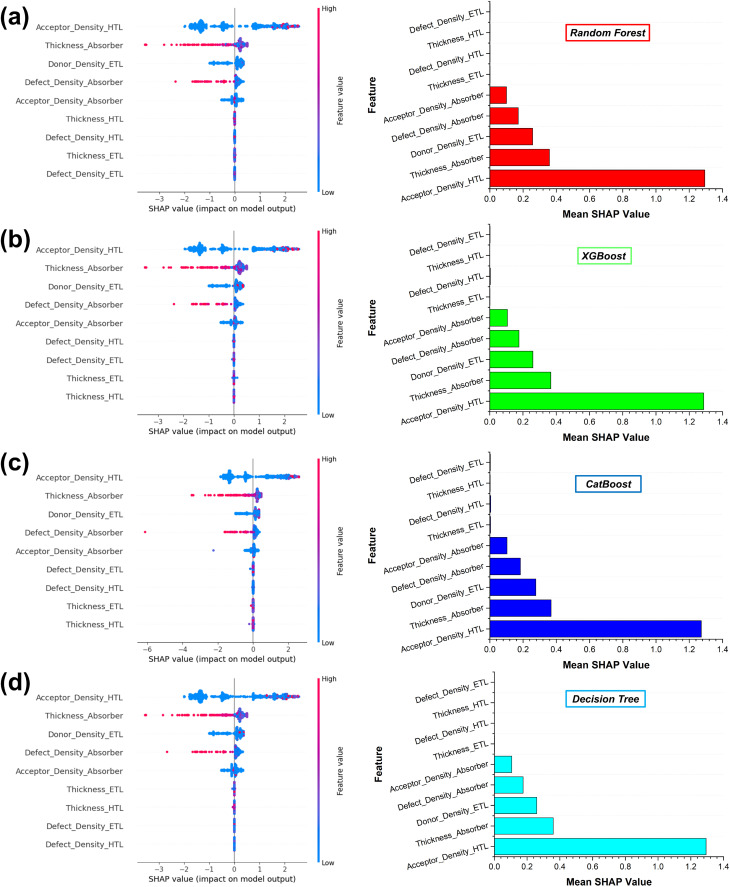
SHAP-based interpretation of feature contributions to PCE (a) RF, (b) XGBoost, (c) CatBoost, and (d) DT.

Fig. 18 illustrates the SHapley Additive exPlanations (SHAP) summary plots for (a) RF, (b) XGBoost, (c) CatBoost, and (d) DT models. All four models provide a transparent and quantitative interpretation of individual device parameters to influence the predicted PCE. Each point represents a sample and is colored according to the feature magnitude (low to high), while the horizontal axis indicates the SHAP value corresponding to the feature's impact on model output. The mean SHAP value for the corresponding features is also represented adjacent to it.

Across all models, *N*_A_ of HTL (Acceptor_Density_HTL) emerges as the most influential parameter for consistently exhibiting the largest SHAP value spread. Higher acceptor densities predominantly contribute positively to PCE enhancement.^3^ Not only that, the magnitude of *N*_A_ is certainly manipulating their critical role in improving hole extraction and reducing interfacial recombination losses. Moreover, a higher *N*_A_ of HTL creates a stronger electric field by enhancing hole extraction efficiency through suppressing electron back-flow into the absorber. Thereby, this phenomenon dramatically reduces interfacial recombination. This dual role makes it the most sensitive parameter for *V*_OC_ and PCE, as confirmed by device physics.^104^ Following this similar pathway, the absorber layer thickness ranks as the second most significant factor, where optimal thickness values positively affect PCE while excessively high values show a diminishing or negative contribution due to increased bulk recombination. The *N*_D_ of the ETL also shows a notable positive influence for ensemble models. This indicates the importance doping profile in facilitating efficient electron transport. Conversely, defect densities within the absorber and transport layers generally exhibit negative SHAP values at higher magnitudes. This is an indicator for confirming that increased trap states adversely affect device performance by promoting non-radiative recombination.

In an important note, CatBoost and XGBoost display smoother and more consistent SHAP distributions compared to the DT model by reflecting their superior interpretability. These findings not only validate the physical relevance of the learned features but also demonstrate that SHAP-assisted machine-learning analysis can reliably bridge data-driven predictions with device physics through offering actionable guidelines for practical solar cell design.

### 4.4. Machine learning performance evaluation

Table 5 mentions the predictive performance of all four supervised learning models, including RF, XGBoost, CatBoost, and DT, while evaluating the model performance with the assistance of Root Mean Squared Error (RMSE), Mean Absolute Error (MAE), *R*^2^-coefficient (*R*^2^), Mean Absolute Percentage Error (MAPE), and accuracy. All these metrics highlight their corresponding model potential for improving the physical structure of the simulated solar cells. The mathematics for the performance evaluator can be written as eqn (19)–(23)19
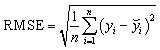
20
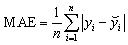
21
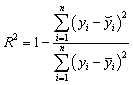
22
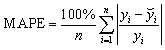
23
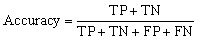
where, *y*_i_, *y̌*_*i*_, *ȳ*_*i*_, *n*, TP, TN, FP, and FN stand for actual/observed value, predicted/forecasted value, mean of actual values, total observation/data point, true positive, true negative, false positive, false negative, respectively.

**Table 5:** Machine learning estimated performance for all four models

Model name	RMSE	MAE	*R* ^2^	MAPE (%)	Accuracy (%)	Max depth	*n* estimators	Learning rate	Depth	Iterations	min samples split
RF	0.243	0.075	0.978	0.397	99.603	10	200				
XGBoost	0.224	0.074	0.982	0.394	99.606	5	100	0.1			
CatBoost	0.209	0.126	0.984	0.656	99.344			0.1	5	200	
DT	0.253	0.090	0.976	0.470	99.530	10					10

Among all models, CatBoost has demonstrated the best overall predictive capability with the lowest RMSE of 0.209 and the highest determination coefficient *R*^2^ of 0.984. These results indicate the superior ability to handle the complex non-linear relationships among the device parameters, likely due to an ordered boosting mechanism and efficient feature interactions.^74^ Despite a slightly higher MAE and MAPE like XGBoost, CatBoost maintains robust generalization with an accuracy of 99.344%. XGBoost ranks second with a strong balance of bias and variance, with an RMSE, MAE, and *R*^2^ value of 0.224, 0.074, and 0.982, respectively. The relatively low error metrics and high accuracy of 99.606% confirms the effectiveness of this model after reaching a maximum depth of 5 with 100 estimators and a learning rate of 0.1. The algorithm XGBoost has already proven by a previous study of U.F. Ali *et al.* in ref. 105 shows a near-perfect *R*^2^ (≈1.0) value for determining the feature importance. The RF model also performs an *R*^2^ value of 0.978 and a low MAE with an accuracy of 99.603%. However, its slightly higher RMSE suggests a limited capability in capturing deeper feature interactions compared to boosting-based algorithms. While RF has shown a marginally higher classification accuracy (99.603%), *R*^2^ and RMSE are the primary metrics for evaluating regression performance. This criterion confirms CatBoost's superiority in predicting the continuous PCE values. Again, the DT model exhibits the weakest performance with the highest RMSE of 0.253 and the lowest *R*^2^ of 0.976 in a maximum depth of 10 and a minimum sample split of 10. This degradation is attributed to overfitting and instability of single tree architectures when modeling higher-dimensional non-linear systems. Overall, the results clearly indicate that ensemble boosting techniques like CatBoost and XGBoost enable high accuracy and reliable prediction of complex PV characteristics.

## 5. Limitations and future perspectives

This numerical study provides valuable insights into the design of efficient KGeCl_3_-based PSC with the simulation approach. However, the SCAPS-1D model employs several simplifying assumptions, including the neglect of ion migration, phase instability, and interfacial chemical degradation.^106^ Those factors are known to critically influence the long-term performance and stability of halide perovskites, particularly for emerging Ge-based compositions. Consequently, the simulated efficiencies reported herein are the theoretical upper limit under ideal and steady-state conditions. Real fabricated devices would likely exhibit lower performance due to multiple loss mechanisms.

To advance this work, future efforts should prioritize experimental validation of KGeCl_3_ thin films. This kind of work helps to predict the optoelectronic properties and stability of the developed cells. Employing more advanced simulation frameworks that incorporate ionic and thermal effects would yield a more realistic performance forecast. Furthermore, interface engineering through passivation layers and the integration of KGeCl_3_ into tandem architectures will make a promising pathway to enhance efficiency and stability.

## 6. Conclusion

A comprehensive study of the lead-free halide perovskite, *i.e.*, KGeCl_3_, as the absorber and CFTS as HTL, integrated with four ETL, namely SnS_2_, PC_60_BM, ZnSe, and WS_2_, has been successfully performed using SCAPS-1D and ML framework. We have made a careful and realistic optimization of the absorber, ETL, and HTL thickness, donor and acceptor densities, defect density properties. Adjacently, the importance of features for performance improvement is determined with the help of four supervised ML algorithms. With the algorithm sets, the highest performance has been marked with the CatBoost algorithm. Additionally, the devices based on WS_2_ showed the highest performance of 21.39%, which was followed by those of ZnSe (21.38%) and PC_60_BM (21.05%). The SnS_2_ devices showed the least efficiency (20.43%). WS_2_ ETL-based structure with the other layer configuration of FTO/CFTS/KGeCl_3_/WS_2_/Au performs best because of its proper band alignment, desirable thickness, and appropriate doping density. The results also suggest that an adequate ETL selection is crucial in improving the PCE of KGeCl_3_-based PSCs. Finally, the outcomes are encouraging, which implies that WS_2_ is a potential ETL to design high-performance lead-free PSCs with high stability, providing a viable pathway for green PV technology.

## Author contributions

**Tanzir Ahamed**: writing – review & editing, writing – original draft, visualization, validation, supervision, software, resources, project administration, methodology, investigation, formal analysis, data curation, conceptualization. **Md. Mehedi Hasan Bappy**: writing – review & editing, resources, methodology, formal analysis. **Mohammad Rahimul Islam**: writing – original draft, visualization, software. **Md. Shihab Uddin**: writing – review & editing, writing – original draft, visualization, validation, software, resources, methodology, formal analysis, data curation, conceptualization. **Md. Arafat Hossain**: writing – review & editing, resources, visualization, data curation. **Tanvir Ahammed**: writing – review & editing, writing – original draft, visualization, validation, software, methodology.

## Conflicts of interest

The authors state that none of the work presented in this study may have been influenced by any known conflicting financial interests or personal ties.

## Data Availability

The data that support the findings of this study are available from the corresponding authors upon reasonable request.
